# Epiplakin attenuates experimental mouse liver injury by chaperoning keratin reorganization

**DOI:** 10.1016/j.jhep.2015.01.007

**Published:** 2015-06

**Authors:** Sandra Szabo, Karl L. Wögenstein, Christoph H. Österreicher, Nurdan Guldiken, Yu Chen, Carina Doler, Gerhard Wiche, Peter Boor, Johannes Haybaeck, Pavel Strnad, Peter Fuchs

**Affiliations:** 1 Department of Biochemistry and Cell Biology, Max F. Perutz Laboratories, University of Vienna, Vienna, Austria; 2 Institute of Pharmacology, Center for Physiology and Pharmacology, Medical University of Vienna, Vienna, Austria; 3 Department of Internal Medicine III and IZKF, University Hospital Aachen, Aachen, Germany; 4 Institute of Pathology, Medical University of Graz, Graz, Austria; 5 Division of Nephrology and Institute of Pathology, RWTH University of Aachen, Aachen, Germany

**Keywords:** PRD, plakin repeat domain, *Eppk1^−/−^*, epiplakin-deficient, K, keratin, CBDL, common bile duct ligation, DDC, 3,5-diethoxycarbonyl-1,4-dihydrocollidine, CCl_4_, carbon tetrachloride, *Krt8^−/−^*, keratin 8-deficient, WT, wild-type, OA, okadaic acid, TMAO, trimethylamine N-oxide, SEM, standard error of the mean, qRT-PCR, quantitative real-time polymerase chain reaction, IHC, immunohistochemistry, ALT, alanine aminotransferase, ALP, alkaline phosphatase, H&E, hematoxylin and eosin, IFM, immunofluorescence microscopy, MDBs, Mallory-Denk bodies, CDCA, chenodeoxycholic acid, TCA, taurocholic acid, Chaperone, Common bile duct ligation, DDC, Keratin aggregates, Plakins

## Abstract

**Background & Aims:**

Epiplakin is a member of the plakin protein family and exclusively expressed in epithelial tissues where it binds to keratins. Epiplakin-deficient (*Eppk1^−/−^*) mice displayed no obvious spontaneous phenotype, but their keratinocytes showed a faster keratin network breakdown in response to stress. The role of epiplakin in the stressed liver remained to be elucidated.

**Methods:**

Wild-type (WT) and *Eppk1*^−/−^ mice were subjected to common bile duct ligation (CBDL) or fed with a 3,5-diethoxycarbonyl-1,4-dihydrocollidine (DDC)-containing diet. The importance of epiplakin during keratin reorganization was assessed in primary hepatocytes.

**Results:**

Our experiments revealed that epiplakin is expressed in hepatocytes and cholangiocytes, and binds to keratin 8 (K8) and K18 via multiple domains. In several liver stress models epiplakin and K8 genes displayed identical expression patterns and transgenic K8 overexpression resulted in elevated hepatic epiplakin levels. After CBDL and DDC treatment, *Eppk1*^−/−^ mice developed a more pronounced liver injury and their livers contained larger amounts of hepatocellular keratin granules, indicating impaired disease-induced keratin network reorganization. In line with these findings, primary *Eppk1*^−/−^ hepatocytes showed increased formation of keratin aggregates after treatment with the phosphatase inhibitor okadaic acid, a phenotype which was rescued by the chemical chaperone trimethylamine N-oxide (TMAO). Finally, transfection experiments revealed that *Eppk1*^−/−^ primary hepatocytes were less able to tolerate forced K8 overexpression and that TMAO treatment rescued this phenotype.

**Conclusion:**

Our data indicate that epiplakin plays a protective role during experimental liver injuries by chaperoning disease-induced keratin reorganization.

## Introduction

Epiplakin is a large protein (>700 kDa) that was originally identified as an autoantigen in the serum of a patient suffering from a subepidermal blistering disease [1]. Encoded by a single exon, epiplakin consists entirely of 13 consecutive plakin repeat domains (PRDs) in humans and 16 repeats in mice [2], [3]. PRDs are a hallmark of proteins belonging to the plakin family which organize the cytoskeleton by binding and interlinking cytoskeletal filaments (for review see [4]). The absence of any other protein domains usually found in plakins makes epiplakin a unique and enigmatic protein.

Expression of epiplakin is restricted to epithelial tissues including simple epithelia of the digestive system [2], [3]. Additional studies revealed that epiplakin binds to intermediate filaments, especially keratins, of simple as well as of stratified epithelia [5], [6]. Surprisingly and in contrast to other plakins, targeted inactivation of epiplakin in mice revealed no obvious *in vivo* phenotype [7], apart from accelerated keratinocyte migration during wound healing [8]. Subsequent *ex vivo* analyses suggested functions for epiplakin in stress response, as epiplakin-deficient (*Eppk1*^−/−^) keratinocytes displayed aggravated keratin disruption upon treatment with phosphatase inhibitors [6]. In addition, *Eppk1*^−/−^ corneal epithelium exhibited increased fragility upon mechanical intervention [9]. Recently, we showed that epiplakin deficiency aggravated experimentally induced pancreatitis [10] indicating a protective role in simple epithelia.

The major binding partners of epiplakin identified so far are keratins including keratin 8 and 18 (K8 and K18), the most frequent keratin heteropolymer expressed in simple epithelia. K8 and K18 play a substantial role in health and disease of simple epithelia, as most comprehensively studied in liver (for review see [11]). During various stress conditions keratin expression is upregulated in liver, which might promote important cytoprotective functions [12]. In adult mice, the only keratins expressed in hepatocytes are K8 and K18, whereas cholangiocytes additionally express K7 and K19 [13].

In the liver of mice, keratin deficiencies such as total lack of K8 or K18 and certain mutations in their genes lead to hepatic phenotypes (for review see [11]). Several human K8 and K18 mutations have been described that predispose transgenic mice to both mechanically- and non-mechanically-induced liver injuries (for review see [11]). K8 and K18 variants identified in human patients predispose to the development and adverse outcome of various liver disorders such as chronic hepatitis C, primary biliary cirrhosis or acute liver failure [11], [14], [15].

Given that epiplakin has not only been shown to be expressed in liver [3], [16] but also to interact with K8/K18 [5] and to be of importance during stress response [6], we aimed to investigate epiplakin’s function in liver under various stress conditions.

For this purpose, we studied the expression of epiplakin upon stress induction and its importance in two liver injury mouse models, i.e. common bile duct ligation (CBDL) and 3,5-diethoxycarbonyl-1,4-dihydrocollidine (DDC) treatment. Additionally, two different *ex vivo* approaches were used to analyze epiplakin’s function during keratin reorganization in primary hepatocytes.

## Materials and methods

### Animal experiments

All animal experiments were in accordance with Austrian Federal Government laws and regulations or approved by the State of Baden-Wurttemberg (Germany) and the University of Ulm (Germany) Animal Care Committee [experimental protocol for carbon tetrachloride (CCl_4_)-induced liver fibrosis development]. The previously described *Eppk1*^−/−^ mice [7] were backcrossed into the C57BL/6J background (N10) and all experiments were performed using age- and sex-matched littermate mice. Additionally, K8-deficient (*Krt8^−/−^*) mice [17] and mice overexpressing human K8 [18] were included in this study. Mice were either subjected to CBDL, a diet supplemented with 0.1% DDC, or CCl_4_-treatment. Details for all mouse injury models are provided in Supplementary methods.

### Cell culture

Primary hepatocytes from wild-type (WT) and *Eppk1*^−/−^ mice were isolated by a two-step collagenase perfusion, enriched by differential centrifugation using a Percoll gradient (Sigma, St Louis, MO) and seeded on collagen-coated dishes. For detailed information on treatment of cells with okadaic acid (OA), transfection experiments and treatment with trimethylamine N-oxide (TMAO), see Supplementary methods.

### Biostatistical analyses

Values of two groups were compared using unpaired two-tailed Student’s *t* test or Mann-Whitney rank sum test. Statistical analyses were performed using GraphPad Prism 5 (GraphPad Software, Inc., La Jolla, CA). *p* values <0.05 were considered statistically significant. Data are expressed as mean ± standard error of the mean (SEM).

### Supplementary methods

Histological, immunohistochemical and immunofluorescence analyses as well as quantitative real-time polymerase chain reaction (qRT-PCR) are described in detail in Supplementary methods. Additionally, comprehensive descriptions of biochemical analyses including preparation of tissue lysates, immunoblotting, blot overlay and pull-down assays along with a detailed list of the antibodies used are provided.

## Results

### Epiplakin is expressed in hepatocytes and cholangiocytes

**Fig. 1:**
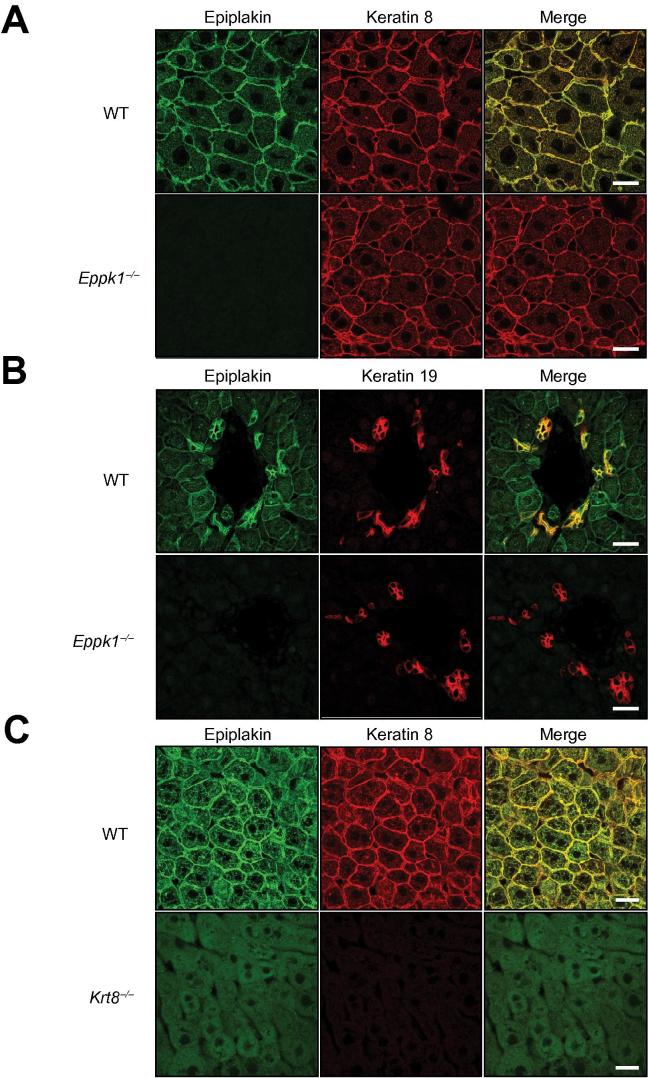
**Epiplakin is expressed in hepatocytes and cholangiocytes, and colocalizes with hepatic K8 and K19.** (A and B) IFM depicting the localization of epiplakin and keratins in murine liver. Immunolabeling of epiplakin (A and B) and K8 (A) or K19 (B) on paraffin sections of livers from WT and *Eppk1*^−/−^ mice. Scale bars: 20 μm. (C) Immunofluorescent labeling of epiplakin demonstrated diffuse protein localization in livers of *Krt8*^−/−^ mice, as opposed to the filamentous epiplakin pattern colocalizing with the keratin network in WT organs (A and B). Scale bars: 20 μm.

In the past, expression of epiplakin in liver was shown to be either confined to hepatocytes [3] or to cholangiocytes with only barely detectable epiplakin levels in hepatocytes [16]. Hence, in order to characterize the localization of epiplakin in liver in more detail, double-immuno-fluorescence stainings for epiplakin and either K8 or K19, the latter representing a specific marker of cholangiocytes, were performed. In livers of WT mice epiplakin was expressed in hepatocytes, whereby its staining pattern closely resembled that of keratin filaments (Fig. 1A). Furthermore, much stronger epiplakin signals than in hepatocytes were detected in cholangiocytes, where the protein colocalized with K19 (Fig. 1B). Accordingly, qRT-PCR analysis revealed that epiplakin mRNA levels were significantly higher in common bile duct and gall bladder than in liver (Supplementary Fig. 1).

### Epiplakin binds to K8 and K18 via multiple domains

Previous studies have demonstrated that nearly all PRDs of epiplakin bind to epidermal keratins [6], [19] whereas up until now only the most C-terminal PRD of human epiplakin has been shown to bind to K8/K18 [5]. Using individual PRDs of mouse epiplakin (Supplementary Fig. 2A), we showed that epiplakin directly binds to K8 and K18 via multiple domains in a blot overlay assay (Supplementary Fig. 2B). These findings were supported by an additional approach by transfecting primary hepatocytes with plasmids coding for individual epiplakin PRD-EGFP-fusion proteins, most of which colocalized with keratins (Supplementary Fig. 2C). The data obtained from blot overlay assays and transfection experiments are summarized in Supplementary material, Supplementary Table 1 and Supplementary Fig. 2D. Additionally, a pull-down assay revealed that soluble K8 co-sedimented with two recombinant PRDs, indicating interaction between soluble keratin subunits and epiplakin (Supplementary Fig. 2E).

To address the importance of keratins for epiplakin distribution, we analyzed the livers of *Krt8^−/−^* mice for their epiplakin staining pattern. In the absence of keratin filaments, a diffuse cytoplasmic localization of epiplakin was observed in hepatocytes (Fig. 1C), indicating that epiplakin does not bind to cytoskeletal filaments other than keratins. Moreover, this experiment demonstrated that in hepatocytes the presence of keratin filaments is a prerequisite for the filamentous epiplakin localization normally found in WT cells.

### *Eppk1*^−/−^ mice display no obvious liver phenotype under physiological conditions

Confirming earlier findings [7], in untreated livers of *Eppk1*^−/−^ mice, no obvious differences in keratin filament organization were detected (Fig. 1A and B). Furthermore, when examining the livers of *Eppk1*^−/−^ mice under physiological conditions, no obvious abnormalities in liver morphology were noted and normal liver enzyme levels were measured in serum of *Eppk1*^−/−^ mice (Table 1). As epiplakin deficiency has been reported to be responsible for reduced expression of the tight junction protein E-cadherin in corneal epithelium [9], we investigated whether cellular junctions were affected in the livers of *Eppk1*^−/−^ mice. However, when analyzing the morphology of desmosomes, adherens junctions and tight junctions using antibodies recognizing the junctional proteins desmoplakin, E-cadherin and occludin, respectively, no irregularities in their localization or staining intensity were detected in unstressed livers of *Eppk1*^−/−^ mice compared to WT livers (Supplementary Fig. 3).

**Table 1:** **Serum liver enzyme levels in WT and *Eppk1*^−/−^ mice under various experimental conditions.**

Abbreviations: ALP, alkaline phosphatase; ALT, alanine aminotransferase; b.d.l., below detection limit. Data are expressed as mean ± SEM. ^∗^*p *<0.05; ^†^*p <*0.005; WT *vs. Eppk1*^−/−^ using unpaired Student’s *t* test or Mann-Whitney rank sum test.

### In experimental liver disease models and in K8-overexpressing mice epiplakin and K8 are upregulated in parallel

**Fig. 2:**
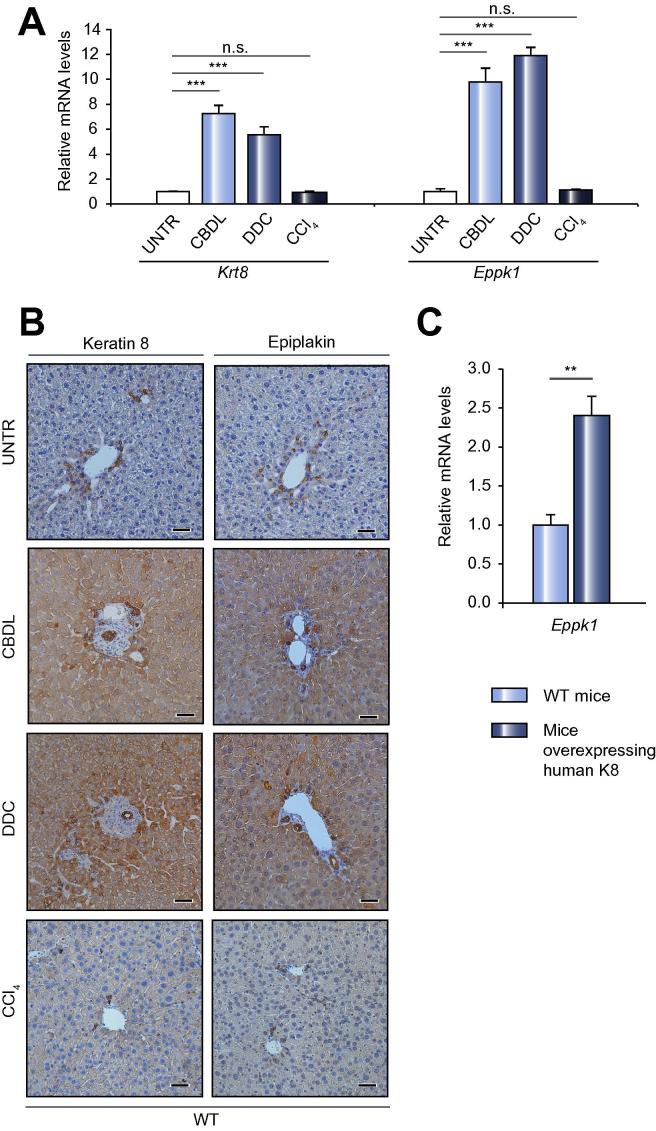
**Parallel upregulation of K8 and epiplakin in livers of mice subjected to various forms of stress and of mice overexpressing human K8.** (A) qRT-PCR analysis demonstrating that upon CBDL and DDC feeding, both *K8* (*Krt8*) and *epiplakin* (*Eppk1*) mRNA levels are increased significantly, whereas CCl_4_ treatment does neither induce upregulation of *K8* nor *epiplakin* mRNA levels. Data are expressed as mean ± SEM; n ⩾4; ^∗∗∗^*p* <0.0005; n.s., not significant. (B) IHC for epiplakin and K8 in healthy, CBDL-, DDC- and CCl_4_-treated livers of WT mice. Scale bars: 100 μm. (C) qRT-PCR analysis demonstrating *epiplakin* mRNA upregulation in liver of mice overexpressing human K8. Data are expressed as mean ± SEM; n ⩾5; ^∗∗^*p* <0.005.

Previous studies have suggested a protective function of epiplakin during keratin filament reorganization in response to cellular stress [6]. Hence, we analyzed whether epiplakin expression in liver is upregulated upon induction of various forms of stress. We therefore quantified *epiplakin* and *K8* mRNA levels in WT livers subjected to CBDL, DDC feeding or CCl_4_-treatment. In line with previous findings [20], [21], *K8* expression was markedly upregulated in WT livers upon CBDL and DDC treatment (Fig. 2A). Strikingly, in both disease models, K8 upregulation was accompanied by strong epiplakin expression, whereas in CCl_4_-treated livers of WT mice neither *epiplakin* nor *K8* mRNA levels were increased (Fig. 2A). Using immunohistochemistry (IHC), we demonstrated that in CBDL- and DDC- but not in CCl_4_-treated livers robust epiplakin upregulation was seen mainly in hepatocytes, closely paralleling the expression pattern of K8 (Fig. 2B). Interestingly, in mice overexpressing human K8 [18], *epiplakin* mRNA levels were also upregulated (Fig. 2C).

### In *Eppk1*^−/−^ mice CBDL results in aggravated liver injury

**Fig. 3:**
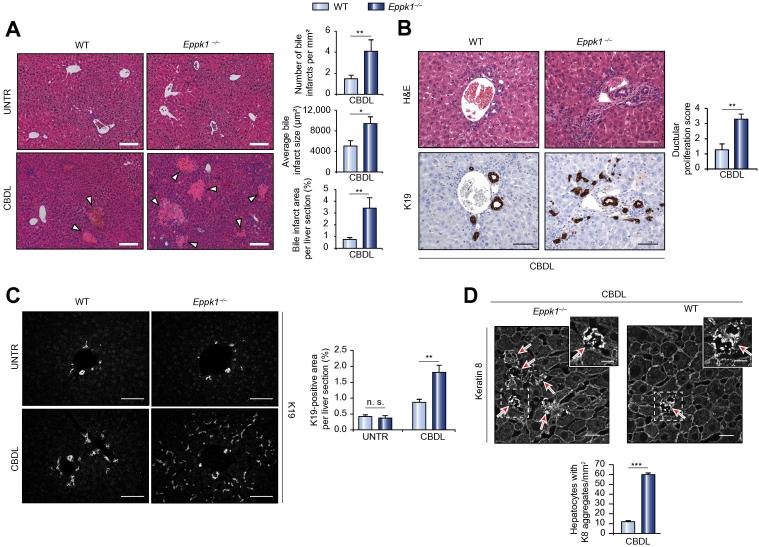
**Epiplakin deficiency aggravates CBDL-induced liver injury and leads to increased formation of keratin aggregates.** (A) Representative H&E stainings and corresponding morphometric analysis of bile infarcts in livers from untreated WT and *Eppk1*^−/−^ mice and mice subjected to CBDL for 5 days. Scale bars: 100 μm; arrowheads depict bile infarcts; UNTR, untreated; data are expressed as mean ± SEM; n ⩾7; ^∗^*p *<0.05; ^∗∗^*p *<0.005. (B) Representative histological and immunohistochemical K19-stainings and corresponding statistical analyses of ductular proliferation score in WT and *Eppk1*^−/−^ mouse livers 5 days after CBDL. Scale bars: 50 μm; data are expressed as mean ± SEM; n ⩾7; ^∗∗^*p *<0.005. (C) Representative IFM for K19 and corresponding morphometric analysis of ductular reaction in livers from untreated WT and *Eppk1*^−/−^ mice and mice subjected to CBDL for 5 days. Scale bars, 100 μm; UNTR, untreated; data are expressed as mean ± SEM; n ⩾7; ^∗∗^*p *<0.005; n.s., not significant. (D) Representative IFM for K8 and corresponding quantification of hepatocytes comprising keratin granules in livers of WT and *Eppk1*^−/−^ mice 5 days after CBDL. Magnifications of areas marked by dashed rectangles are displayed in the upper right corner of the respective panels. Scale bars: 20 μm; arrows depict hepatocytes comprising keratin granules; data are expressed as mean ± SEM; n ⩾7; ^∗∗∗^*p *<0.0005.

To test whether the strong upregulation of epiplakin expression upon CBDL was indicative of a protective function of the protein during obstructive cholestasis, WT and *Eppk1*^−/−^ mice were subjected to CBDL. Five days after surgery, alanine aminotransferase (ALT) levels were significantly increased in serum from *Eppk1*^−/−^ mice compared to their littermate controls (Table 1). In addition, *Eppk1*^−/−^ mice showed elevated serum levels of bilirubin and alkaline phosphatase (ALP), however, no statistical significance was found (Table 1). Moreover, quantitative histological evaluation of hematoxylin and eosin (H&E)-stained liver sections revealed significantly increased numbers and sizes of bile infarcts, resulting in considerably larger areas of necrotic tissue in *Eppk1*^−/−^ livers in comparison to their WT counterparts (Fig. 3A).

Next, we analyzed the extent of bile duct proliferation, a regenerative response to liver injury. Ductular reaction was assessed by histo-pathological evaluation of paraffin sections stained with H&E or immunostained for K19 (Fig. 3B), morphometric analysis of K19-positive areas on liver sections (Fig. 3C), and immunoblot analysis of K19 levels in total liver protein lysates from WT and *Eppk1*^−/−^ mice (Supplementary Fig. 4A). All experiments demonstrated significantly increased numbers of ductular proliferates as well as higher K19 levels in *Eppk1*^−/−^ CBDL livers compared to WT controls, indicating aggravated liver injury in *Eppk1*^−/−^ mice.

### *Eppk1*^−/−^ livers display impaired keratin reorganization upon CBDL

Mice lacking or overexpressing hepatic keratins are predisposed to liver disorders development [17], [22], [23], [24]. Thus, to investigate a potential impact of epiplakin on keratin expression, we compared total keratin levels in WT and *Eppk1*^−/−^ livers. When total liver lysates were analyzed by immunoblotting, equal levels of K8 and K18 were revealed in WT and *Eppk1*^−/−^ livers, in untreated mice, and in mice subjected to CBDL (Supplementary Fig. 4B and C). Thus, it was excluded that the aggravated liver injury observed in *Eppk1*^−/−^ mice was caused by aberrant keratin expression.

The parallel upregulation of epiplakin and hepatic keratins upon biliary obstruction suggested a role of epiplakin in the organization of keratin networks during a stress response. Therefore, we examined the keratin network in CBDL livers of WT and *Eppk1*^−/−^ mice. When studying the K8/K18 filament organization using immunofluorescence microscopy (IFM), we observed not only increased density of the keratin network as described previously for WT mice undergoing CBDL [20], but also irregular keratin patterns clearly resulting from the formation of bile infarcts. Additionally, in non-necrotic areas, we noticed single hepatocytes comprising aggregated keratin structures (Fig. 3D). While keratin granules were also detectable in WT hepatocytes, these keratin aggregations were significantly more frequent in *Eppk1*^−/−^ livers (Fig. 3D).

### In *Eppk1*^−/−^ mice DDC treatment results in aggravated liver injury

In WT mice subjected to a DDC diet, epiplakin and K8 were both strongly upregulated (Fig. 2A and B). Hence, in order to analyze whether lack of epiplakin increases the susceptibility of mice to DDC-induced liver injury, livers of WT and *Eppk1*^−/−^ mice were challenged via administration of DDC. After four weeks of DDC feeding, *Eppk1*^−/−^ mice displayed significantly elevated ALT serum levels compared to WT mice (Table 1), indicating a more severe course of liver disease. In contrast, bilirubin and ALP serum levels were comparable in sera of WT and *Eppk1*^−/−^ mice (Table 1), suggesting that epiplakin deficiency increased the susceptibility of hepatocytes to DDC intoxication rather than aggravated cholestatic injury.

**Fig. 4:**
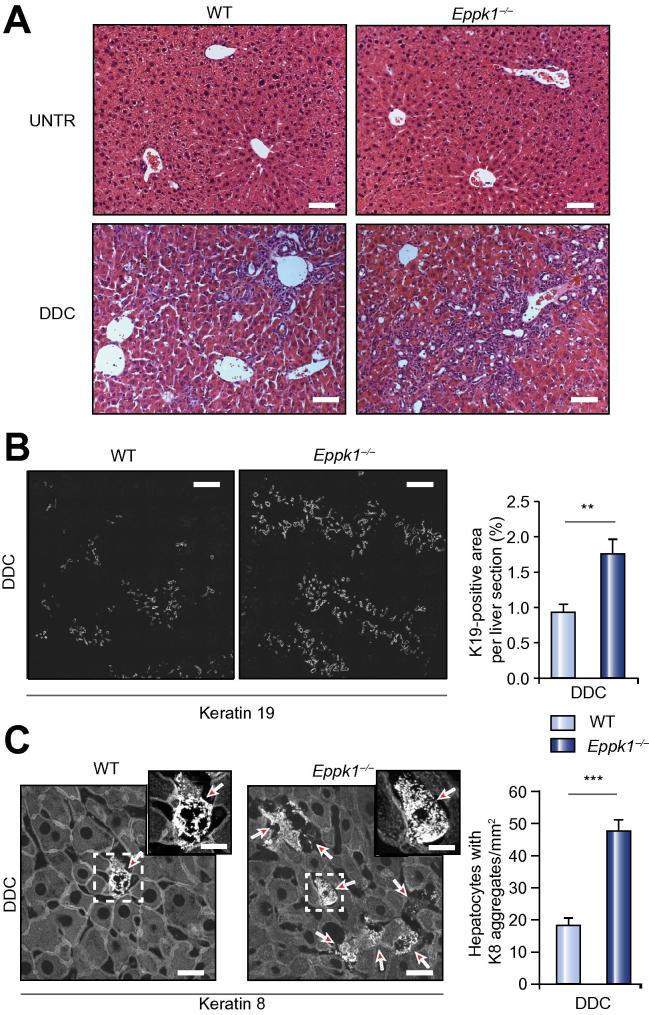
E**piplakin deficiency aggravates DDC-induced liver injury and leads to increased formation of keratin aggregates.** (A) Representative H&E stainings of liver sections from untreated and DDC-treated (4 weeks) WT and *Eppk1*^−/−^ mice. Scale bars, 100 μm. UNTR, untreated. (B) Representative IFM for K19 and corresponding morphometric analysis of bile duct mass. Scale bars: 100 μm; data are expressed as mean ± SEM; n ⩾6; ^∗∗^*p* <0.01. (C) Representative IFM for K8 and corresponding quantification of hepatocytes comprising keratin granules in livers of WT and *Eppk1*^−/−^ mice after DDC treatment. Magnifications of areas marked by dashed rectangles are displayed in the upper right corner of the respective panels. Scale bars: 20 μm; arrows depict hepatocytes comprising keratin granules; data are expressed as mean ± SEM; n ⩾6; ^∗∗∗^*p *<0.0001.

In *Eppk1*^−/−^ livers, increased ductular proliferation was noted during examination of H&E-stained DDC livers (Fig. 4A). Using morphometric analysis of the bile duct mass (Fig. 4B) as well as immunoblot analysis of K19 levels in protein lysates (Supplementary Fig. S5A) of livers from WT and *Eppk1*^−/−^ mice, significantly larger numbers of ductular proliferates or higher K19 levels, respectively, were detected in *Eppk1*^−/−^ DDC livers than in WT controls.

### *Eppk1*^−/−^ livers display impaired keratin reorganization after DDC treatment

Next, using immunoblot analysis, we investigated whether after four weeks of DDC administration hepatic keratin expression was modified in *Eppk1*^−/−^ livers compared to WT controls. K8 and K18 levels were markedly increased after DDC feeding in livers from both WT and *Eppk1*^−/−^ mice, but no differences between both genotypes were observed (Supplementary Fig. 5B and C). Subsequently, we examined immunofluorescently labeled K8 on paraffin sections obtained from DDC-treated WT and *Eppk1*^−/−^ livers. Consistent with our observations made during analysis of CBDL livers, significantly more cells comprising keratin granules were detected in DDC-treated *Eppk1*^−/−^ livers than in their WT counterparts (Fig. 4C).

Aggregates containing K8/K18 are a common pathological feature of different liver disorders and are most commonly referred to as Mallory-Denk bodies (MDBs) (for review see [25]). Thus, using IFM, we analyzed whether the K8 aggregations found in CBDL- and DDC-treated livers comprised protein markers typical for MDBs. All keratin aggregates detected in CBDL- and DDC-treated livers of both genotypes were negative for ubiquitin, p62 and Hsp70 (Supplementary Fig. 6), and therefore do not correspond to MDBs.

### Hepatocytes comprising keratin aggregates found in CBDL- and DDC-treated livers are dying

IFM analysis of liver paraffin sections derived from WT and *Eppk1*^−/−^ mice subjected to CBDL or DDC treatment revealed loss of nuclei in a vast majority of hepatocytes comprising K8 aggregates, indicating that these cells were dying (Supplementary Fig. 7). As all of these hepatocytes were negative for cleaved caspase-3 (Supplementary Fig. 8), the death of cells comprising aggregated keratins is likely caused by necrosis rather than by activation of apoptotic pathways.

### WT and *Eppk1*^−/−^ hepatocytes show comparable susceptibility to bile acid-induced cytotoxicity

To analyze the susceptibility of WT and *Eppk1*^−/−^ hepatocytes to toxic bile acids as occurring upon local bile leakage into the parenchyma, we incubated primary hepatocytes isolated from WT and *Eppk1*^−/−^ livers with chenodeoxycholic acid (CDCA) or taurocholic acid (TCA). Surprisingly, *Eppk1*^−/−^ hepatocytes were equally susceptible to bile acid exposure as their WT counterparts (Supplementary Fig. 9A). However, in contrast to CBDL- and DDC-treated livers, no elevation of keratin or epiplakin expression levels and, consequently, no keratin aggregation was detected in WT or *Eppk1*^−/−^ hepatocytes upon bile acid exposure (Supplementary Fig. 9B and C).

### *Eppk1*^−/−^ hepatocytes display impaired keratin filament reorganization upon OA treatment which can be prevented by the chemical chaperone TMAO

In order to verify that epiplakin is required during stress-induced keratin filament reorganization, primary hepatocytes were treated with the phosphatase inhibitor OA, which leads to hyperphosphorylation of keratins and thereby affects keratin filament organization [26], [27]. Although total keratin levels are not increased in this stress model, OA treatment strongly elevates the pool of cytosolic keratins [26], [28] associated with the disassembly of keratin filaments [26], [27].

**Fig. 5:**
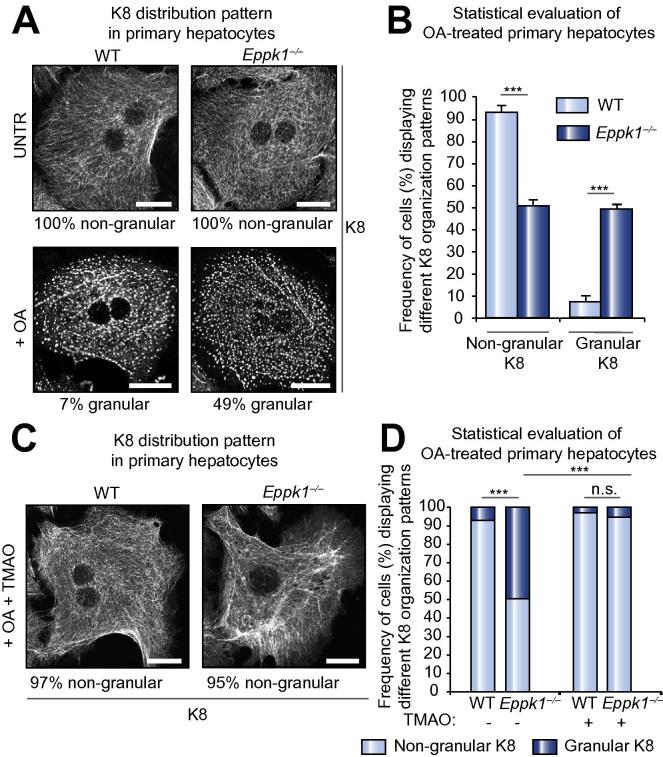
**Lack of epiplakin leads to impaired OA-induced keratin reorganization in primary hepatocytes, which is prevented by TMAO treatment.** (A) Representative images of untreated and OA-treated hepatocytes displaying either non-granular or granular K8 staining patterns, respectively. Frequency of the presented staining pattern is indicated below each image. Scale bars: 20 μm. (B) Statistical analysis of the frequency of hepatocytes displaying either of the two keratin network organization patterns shown in (A). Data are expressed as mean ± SEM; n ⩾2492 cells from 3 individual isolations; ^∗∗∗^*p *<0.0005. (C) Representative images of WT and *Eppk1*^−/−^ hepatocytes treated with OA and TMAO. Frequency of the presented staining pattern is indicated below each image. Scale bars: 20 μm. (D) Statistical evaluation of the proportional distribution of cells displaying granular or non-granular keratin network organization upon OA treatment in the absence or presence of TMAO. Data are expressed as mean ± SEM; n ⩾2793 cells from 3 individual isolations; ^∗∗∗^*p <*0.0005; n.s., not significant.

After exposure of primary hepatocytes to OA, the number of cells displaying either non-granular or granular keratin network organization was assessed (Fig. 5A). *Eppk1*^−/−^ hepatocytes were found significantly more often to exhibit granular keratins than WT cells (Fig. 5A and B). Immunoblot analyses revealed that the increased keratin network disruption in OA-treated *Eppk1*^−/−^ hepatocytes was not caused by alterations of the keratin phosphorylation status (Supplementary Fig. 10).

Interestingly, OA-treated WT and *Eppk1*^−/−^ cells which were subsequently exposed to bile acids, displayed similar cell death rates, indicating that increased occurrence of keratin aggregates does not render hepatocytes more susceptible to bile acid-induced cytotoxicity (data not shown).

Recently, low molecular weight chemical chaperones have been shown to protect proteins from aggregating, among them K5/K14 [29]. Hence, we investigated whether the chaperone TMAO ameliorates the enhanced OA-induced formation of keratin aggregates observed in *Eppk1*^−/−^ hepatocytes. For this purpose, cultivation and OA treatment of hepatocytes were performed in the presence of TMAO. The chaperone was found to significantly reduce the number of *Eppk1*^−/−^ hepatocytes comprising K8 granules by increasing the amount of cells displaying non-granular keratin (Fig. 5C and D).

### *Eppk1*^−/−^ hepatocytes show reduced tolerance for forced K8 overexpression which can be rescued by TMAO

**Fig. 6:**
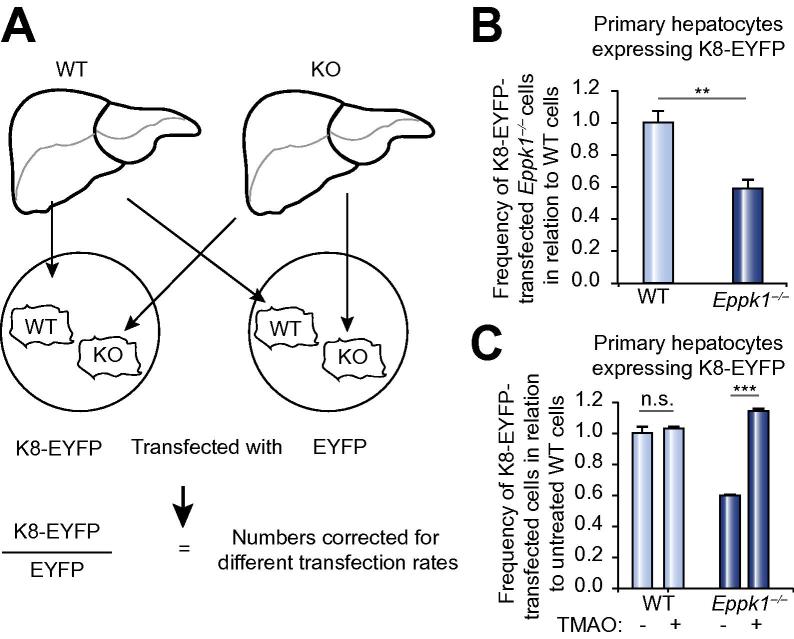
***Eppk1*^−/−^ hepatocytes show reduced tolerance for forced K8 expression, which is rescued by TMAO treatment.** (A) Schematic overview showing the design of primary hepatocytes transfection experiments. WT and *Eppk1*^−/−^ hepatocytes were co-cultivated and transfected with a plasmid encoding K8-EYFP or EYFP, followed by counting of K8-EYFP- and EYFP-positive cells. To correct for variations in transfection rate between WT and *Eppk1*^−/−^ hepatocytes in individual isolations, the number of K8-EYFP-positive cells was divided by the number of EYFP-positive cells for each single experiment. (B) Significantly less *Eppk1*^−/−^ than WT hepatocytes were found to express EYFP-tagged K8, indicating difficulties of *Eppk1*^−/−^ cells to tolerate high K8 levels. The WT mean was arbitrarily set to 1. Data are expressed as mean ± SEM; n ⩾140 cells from 6 individual isolations; ^∗∗^*p *<0.005. (C) Comparison of K8-EYFP-transfected WT and *Eppk1*^−/−^ hepatocyte numbers cultivated in the absence or presence of TMAO. The untreated WT mean was arbitrarily set to 1. Data are expressed as mean ± SEM; n ⩾123 cells from 3 individual isolations; ^∗∗∗^*p <*0.0001; n.s., not significant.

In order to analyze the effect of elevated K8 levels on hepatocytes as seen during CBDL and DDC treatment, keratin reorganization in primary hepatocytes was challenged by forced overexpression of K8. Therefore, WT and *Eppk1*^−/−^ cells were co-cultured and transfected with a plasmid coding for K8 fused to EYFP or the empty EYFP vector as a control (Fig. 6A). Remarkably, significantly fewer *Eppk1*^−/−^ hepatocytes were found to express K8-EYFP than their WT counterparts (Fig. 6B), suggesting difficulties of *Eppk1*^−/−^ hepatocytes to cope with high K8 levels.

In transfected hepatocytes, the newly synthesized K8-EYFP proteins were either integrated in filaments (non-granular) or accumulated as keratin granules (Supplementary Fig. 11A). In line with data from experiments with OA, treatment with TMAO increased the number of *Eppk1*^−/−^ hepatocytes comprising non-granular K8-EYFP (Supplementary Fig. 11B and C). Next, we assessed whether this effect of TMAO was also able to ultimately rescue the diminished total number of *Eppk1*^−/−^ hepatocytes tolerating high K8-EYFP levels. Quantification of WT and *Eppk1*^−/−^ cells expressing K8-EYFP demonstrated that significantly more K8-EYFP-expressing *Eppk1*^−/−^ cells were found in the presence of TMAO than in its absence (Fig. 6C).

Taken together, these data showed that TMAO was able to elevate the total number of *Eppk1*^−/−^ hepatocytes expressing K8-EYFP, which was achieved mainly by increasing the amount of cells displaying a filamentous K8-EYFP pattern (summarized in Supplementary Fig. 11C). These findings demonstrated that the aggregation-preventing properties of TMAO rescued the phenotype observed in *Eppk1*^−/−^ hepatocytes by enabling them to tolerate high keratin levels and, potentially, to avoid cell death resulting from impaired keratin reorganization.

## Discussion

Our data show that *Eppk1*^−/−^ mice display no obvious abnormalities in liver, which is in line with previous findings [7]. Under physiological conditions, lack of epiplakin does not affect the keratin filament organization in hepatocytes and the localization of major cell-cell junction proteins is unaltered. However, after CBDL and DDC treatment, *Eppk1*^−/−^ mice developed a more pronounced liver injury. Differences in inflammatory response are unlikely to be the primary cause of this phenotype since inflammatory cells do not express epiplakin and no indications for enhanced inflammatory cell infiltration in stressed *Eppk1*^−/−^ livers were found (data not shown). Furthermore, we currently have no evidence that increased biliary rupture contributes to the phenotypes observed in *Eppk1*^−/−^ mice. Moreover, our data suggest that *Eppk1*^−/−^ hepatocytes are not more susceptible to cytotoxicity conferred by the mere presence of toxic bile acids, but rather that epiplakin exerts its protective function during stress-induced keratin upregulation and consequent rearrangement of the keratin network.

Therefore, we propose that during certain stress conditions epiplakin protects from aggravated liver injury by interacting with hepatic keratins which are established stress-protective proteins [12]. This hypothesis is supported by several observations: i) epiplakin perfectly colocalizes with K8/K18 filaments in hepatocytes; ii) most of epiplakin’s 16 PRDs are able to bind to K8/K18; iii) in several liver stress models, hepatic epiplakin and K8 are co-regulated on the transcriptional level; iv) the filament-associated localization of epiplakin in hepatocytes is completely dependent on K8/K18 as shown in cells devoided of keratin filaments; v) upon experimental injury, livers from *Eppk1*^−/−^ mice contained increased numbers of hepatocytes comprising keratin granules.

**Fig. 7:**
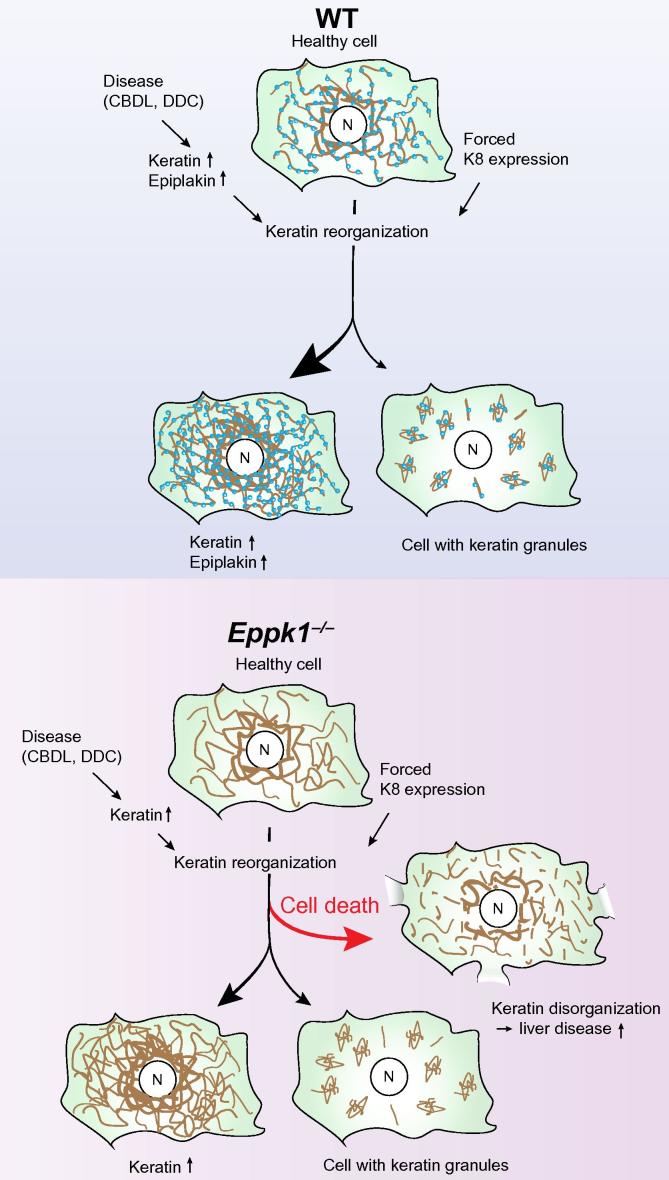
**Loss of epiplakin impairs stress-induced keratin reorganization leading to aggravated experimental liver injury.** The scheme visualizes a model of epiplakin’s role during disease-induced or forced keratin upregulation and consequent filament reorganization in hepatocytes. In untreated WT cells, epiplakin (blue) colocalizes with keratin filaments (brown). Under physiological conditions (healthy cell), epiplakin deficiency does not cause alterations in keratin network organization. In hepatocytes of both genotypes, keratin network reorganization is triggered by experimental liver disease-induced keratin upregulation or by forced keratin expression. *Eppk1*^−/−^ cells suffer from keratin disorganization which leads to cell death further aggravating liver disease. In contrast to *Eppk1*^−/−^ hepatocytes, parallel upregulation of epiplakin enables WT cells to tolerate high keratin protein levels by successfully reorganizing their keratin filament networks. N, nucleus.

How does epiplakin’s interaction with keratins protect from experimental liver disease? In stressed liver, keratin expression is typically increased up to 3-fold leading to new filament formation and strengthening of existing filaments, which is thought to promote the important cytoprotective functions provided by K8 and K18 [12]. Keratins represent about 0.3% of total cellular protein in liver [30]. Given the strong upregulation of keratins during certain liver disorders, this already high amount is significantly increased within a very short time frame and therefore possibly challenging the cellular systems ensuring keratin proteostasis. In this respect, overexpression of several intermediate filaments including glial fibrillary acidic protein, peripherin or neurofilaments in mice has been shown to be toxic, leading to neurological disorders affecting the respective organs [31]. In contrast, high keratin levels in liver per se are generally well tolerated under basal conditions [12]. However, elevated keratin levels are a co-factor for liver injury as shown by K8 overexpressing mice fed with a high fat diet, which showed accumulation of K8/K18 and increased keratin misfolding [24]. This led us to speculate that disease-induced keratin overexpression without parallel protective upregulation of epiplakin leads to aggravated liver injury caused by impaired keratin network reorganization and subsequent hepatocellular death. A scheme depicting this model is shown (Fig. 7). This hypothesis is strongly supported by our data showing that the chemical chaperone TMAO rescues the diminished number of *Eppk1*^−/−^ hepatocytes artificially overexpressing K8. TMAO has been shown to be capable of decreasing the accumulation and aggregation of different proteins [32], [33]. In line with that, TMAO was shown to significantly reduce the number of keratin aggregate-containing keratinocytes derived from patients suffering from epidermolysis bullosa simplex [29]. The fact that this chemical chaperone was able to rescue two different phenotypes observed in primary *Eppk1*^−/−^ hepatocytes strongly indicates that epiplakin fulfills important keratin chaperone-like functions in liver.

The importance of proteins chaperoning K8/K18 for cellular homeostasis was also demonstrated by studies on the co-chaperone Mrj (Mammalian relative of DnaJ), showing that its absence in chorionic trophoblast cells prevents chorioallantoic attachment during placental development [34]. Similar to stressed *Eppk1*^−/−^ hepatocytes Mrj-deficient chorionic trophoblast cells displayed keratin aggregates.

Recently, we reported that *Eppk1*^−/−^ mice suffered from aggravated experimentally-induced pancreatitis, and more of their acinar cells displayed keratin aggregates [10]. Together with the new findings presented in this work these data suggest a general function of epiplakin in chaperoning keratins in several organs comprising of simple epithelia. In contrast, the function of epiplakin in stratified epithelia seems to be slightly different, as indicated by studies showing enhanced migratory potential of *Eppk1*^−/−^ keratinocytes during wound healing [8] and increased fragility of corneal epithelium upon mechanical intervention [9]. These findings are not surprising given that epiplakin binds to different keratins and that their functions are considered to be multifactorial and tissue-specific [11].

The proposed role of epiplakin in chaperoning keratins is remarkably different from those of most other members of the plakin protein family. In mice, targeted inactivation of epiplakin showed no obvious phenotype, whereas mice deficient for other plakins (e.g. plectin, desmoplakin, bullous pemphigoid antigen 1, and microtubule-actin cross-linking factor 1) revealed severe phenotypes including early death, skin blistering, and muscle weakness (for review see [4]). These findings demonstrate a role of these plakins in ensuring cellular and tissue integrity upon mechanical stress. The entirely different function of epiplakin is possibly based on its exceptional structure comprising solely PRDs, most of which bind to keratins, whereas many other plakins interconnect different cytoskeletal filament systems and cell junction complexes via specific binding domains. Thus, epiplakin is an unusual plakin with regards to both its structure and function.

Further studies are needed to elucidate the precise mechanisms of how epiplakin chaperones the reorganization of keratin networks and whether these protective functions extend to other tissues and/or stress situations. In addition, genetic studies could reveal whether the absence of epiplakin or mutations in its gene are general risk factors for complications in other pathologies of the gastrointestinal tract.

## Financial support

This work was supported by grant P 22604-B12 from the 10.13039/501100002428Austrian Science Fund (FWF) (to PF), grant STR 1095/4-1 from the German Research Foundation and the Interdisciplinary Center for Clinical Research (IZKF) in Aachen (to PS) and grant SFB/Transregio 57 DFG consortium ‘‘Mechanisms of organ fibrosis’’ from the German Research Foundation and the Interdisciplinary Center for Clinical Research (IZKF) in Aachen (to PB and PS). The funders had no role in study design, data collection and analysis, decision to publish, or preparation of the manuscript.

## Conflict of interest

The authors who have taken part in this study declared that they do not have anything to disclose regarding funding or conflict of interest with respect to this manuscript.

## Author’s contributions

Study concept and design: PF.

Acquisition of data: SS, KLW, CHÖ, NG, YC, CD, PB, PF.

Analysis and interpretation of data: SS, KLW, PB, JH, PS, PF.

Drafting of the manuscript: SS, KLW, PF.

Critical revision of the manuscript for important intellectual content: all authors.

Statistical analysis: SS, KLW.

Obtained funding and study supervision: PF.

Technical or material support: JH, GW, PF.
