# Supplementary material for: Epiplakin attenuates experimental mouse liver injury by chaperoning keratin reorganization
Source: J Hepatol. 2015 Jun;62(6):1357–66. doi: 10.1016/j.jhep.2015.01.007 (PMC4451473; doi:10.1016/j.jhep.2015.01.007)
Supplement: Supplementary data [file mmc1.pdf]

Supplementary data to

Epiplakin attenuates experimental mouse liver injury by chaperoning keratin reorganization.

Sandra Szabo, Karl L. Wögenstein, Christoph H. Österreicher, Nurdan Guldiken, Yu Chen, Carina Doler, Gerhard Wiche, Peter Boor, Johannes Haybaeck, Pavel Strnad, Peter Fuchs

Table of Contents

Supplementary Methods 3
- Mouse injury models 3
- Tissue preparation, histology and immunostainings 4
- Histological evaluations 4
- Image acquisition 5
- Quantitative real-time PCR 5
- Liver protein lysates 6
- Coomassie staining and immunoblotting 7
- Expression and purification of recombinant proteins 7
- Blot overlay of liver keratins with plakin repeat domains 7
- Keratin PRD-GST Pulldown 8
- Antibodies 8
- Isolation and cultivation of primary hepatocytes 9
- Bile acid treatment of primary hepatocytes 9
- Okadaic acid treatment of primary hepatocytes 9
- Overexpression of fluorescently tagged proteins in primary hepatocytes 10

Supplementary Figures 12
- Fig. S1. Epiplakin transcript levels are higher in the biliary epithelium than in the liver 12
- Fig. S2. Epiplakin interacts with hepatic keratins via multiple plakin repeat domains 13

Fig. S3. Localization of the cell-cell junctional proteins desmoplakin, e-cadherin and occludin is unaltered in Eppk1─/─ livers 15
Fig. S4. Immunoblot analyses of CBDL-induced liver injury in WT and Eppk1-/- mice. 16
Fig. S5. Immunoblot analyses of DDC-induced liver injury in WT and Eppk1─/─ mice......18
Fig. S6. Keratin aggregates formed after CBDL and DDC feeding do not colocalize with ubiquitin, p62 or Hsp70. 20
Fig. S7. Hepatocytes comprising keratin aggregates found in CBDL- and DDC-treated livers predominantly display loss of nuclei...22
Fig. S8. Death of hepatocytes comprising keratin aggregates is not caused by activation of apoptotic pathways 24
Fig. S9. Lack of epiplakin does not influence susceptibility of hepatocytes to bile acid exposure...26
Fig. S10. Okadaic acid treatment results in comparable levels of phosphorylated keratins in WT and Eppk1─/─ hepatocytes. 28
Fig. S11. TMAO treatment rescues the diminished number of Eppk1─/─ hepatocytes displaying a non-granular K8-EYFP pattern 29
Table S1. Summary of findings obtained from blot overlay and transfection experiments .31
Supplementary References 32

Supplementary Methods

Mouse injury models*.* All animals received humane care, were kept under standardized conditions and had free access to water and food. To study the effect of CBDL on WT and *Eppk1*$^{-/-}$ mice, animals were anaesthetized with ketamine and xylazine. After midline laparotomy, the common bile duct was exposed and double ligated with 6-0 silk sutures. The abdomen was closed in layers, and the animals were allowed to recover on a heat pad. After 5 days, mice were anaesthetized with 150 µl isoflurane and killed by cervical dislocation. Successful bile duct ligation was evaluated by macroscopic evaluation and an increased, dilated gallbladder. To study the effects of DDC-feeding in WT and *Eppk1*$^{-/-}$ mice, mice were fed with a diet supplemented with 0.1% DDC (#137030, Sigma-Aldrich, St Louis, MO) for 4 weeks. Control animals were exposed to the corresponding DDC-free diet for the same period. After 4 weeks, animals were sacrificed for collection of blood and liver samples.

To study the effect of an additional liver stress model on epiplakin and keratin upregulation, WT mice were injected intraperitoneally twice a week for 12 weeks with 0.2 ml CCl_4/kg mouse weight diluted in olive oil (both from Sigma) and sacrificed 96 hours after the last injection. Mice were killed using CO_2 inhalation and livers were removed for RNA isolation.

Serum levels of liver enzymes were measured at Invitro laboratories (Invitro, Vienna, Austria). Livers were isolated and pre-defined lobes were either frozen in liquid nitrogen for biochemical analyses or cryo-sectioning, respectively, or routinely fixed in 4% paraformaldehyde for histology and immunostainings.

Tissue preparation, histology and immunostainings. Paraformaldehyde-fixed tissues were embedded in paraffin, sectioned (2–5 µm) and stained with H&E. For IHC and IFM, sections were subjected to antigen retrieval by boiling them in EDTA buffer. Blocking of endogenous peroxidases and unspecific antigen interactions was performed with 3% H_2O_2 and 2% BSA, respectively. Afterwards, sections were incubated with primary antibodies for 1 hour at room temperature. Fluorophore-conjugated antibodies were used for IFM. Antibodies in IHC were visualized by biotinylated secondary antibodies and diaminobenzidine (DAB) detection using the Liquid DAB+ Substrate Chromogen System (Dako, Glostrup, Denmark). Additionally, for K19 IHC on CBDL liver sections, paraffin sections were incubated with Epitope Retrieval Solution, pH 6.0 (Dako), at 98.5°C for 40 minutes, followed by cooling at room temperature for 20 minutes. IHC was performed on an automatic immunostainer (Dako) and detection was accomplished using REAL TM Envision+ DAB (Dako).

Histological evaluations. Serial sections of CBDL-livers from WT and *Eppk1*$^{-/-}$ animals were stained with H&E and immunostained with antibodies recognizing K19. Necrotic areas in liver tissue (bile infarcts) were measured and quantified using Axiovision Rel. 4.8 software (Carl Zeiss Inc., Jena, Germany). For analysis of ductular proliferation, H&E- as well as K19-immunostainings were individually evaluated for ductular reaction by a trained pathologist (J.H.) in a blinded manner. Scoring was performed using a scale from 0–4, reflecting none to massive bile duct proliferation (0, none; 1, rare; 2, moderate; 3, frequent; 4, massive). In a second approach, for quantification of the bile duct mass, liver paraffin sections derived from CBDL- and DDC-treated mice were immunofluorescently labeled with antibodies recognizing K19 and subjected to image acquisition. On each liver paraffin section,

4.5 mm^2 were scanned for the presence of K19-positive cells. Subsequently, the K19-positive areas were quantified using Icy software (Quantitative Image Analysis Unit, Institut Pasteur, France). For quantification of hepatocytes comprising keratin aggregates, liver paraffin sections derived from CBDL- and DDC-treated mice were labeled with a K8 antibody and subjected to IFM. On each liver paraffin section, 4.5 mm^2 were scanned for the presence of K8 aggregates.

Image acquisition. IFM was performed using a confocal laser-scanning microscope (Zeiss LSM 710) equipped with Plan-Apochromat 40x/1.4 NA oil-immersion objective lenses, using the ZEN software (Carl Zeiss Inc.). H&E as well as IHC images were acquired with an Axiophot microscope (Carl Zeiss Inc.) equipped with either Plan-Neofluar 10x/0.3 NA or Plan-Neofluar 20x/0.5 NA objectives using AxioVision 4.8 software (Carl Zeiss Inc.). Further image adjustments (brightness and contrast) were accomplished using Photoshop CS5 (Adobe Systems, San Jose, CA) or ImageJ software (National Institutes of Health).

Quantitative real-time PCR. Livers, common bile ducts and gall bladders were isolated and submerged into RNAlater™ Stabilization Reagent (Ambion, Life Technologies GmbH, Darmstadt, Germany) for RNA analysis. Total RNA was isolated using RNeasy mini kit (Qiagen, Valencia, CA) and converted into cDNA using Superscript II reverse transcriptase (Invitrogen, Carlsbad, CA) and the oligo-dT method. Quantitative real-time PCR was performed with a 7500 fast Real Time PCR Sequence Detection System (Applied Biosystems, Foster City, USA). Samples were analyzed in duplicates and ribosomal protein L7 was employed as an internal standard (primers: 5’-GAAAGGCAAGGAGGAAGCTCATCT-3’ and 5’-AATCTCAGTGCGGTACATCTGCCT-3’). The transcript levels of epiplakin as well as

human K8 relative to the internal control were determined using primers specific for murine epiplakin (5’-ATGGGTACCCCTGGTTTTTC-3’ and 5’-CAGGGTGTGGAAAGTGGTCT-3’) and human K8 (5’-GCTGACCGACGAGATCAACT-3’ and 5’-CATGGACAGCACCACAGATG-3’), respectively.

Liver protein lysates. For preparation of total liver protein lysates, livers were dissected and snap frozen in liquid nitrogen. The tissue was then ground in a mortar and homogenized in lysis buffer [10 mM Tris pH 7.5; 150 mM NaCl; 5 mM $MgCl_2$; 5 mM EDTA; 1% Triton X-100; 0.1% SDS; 1% deoxycholate; Protease Inhibitor Cocktail (Roche Applied Science, Indianapolis, IN); Phosphatase Inhibitor Cocktails (Sigma-Aldrich), 0.5 mg/ml DNAse I; 0.2 mg/ml RNAse A; 1 mM PMSF] using a Dounce tissue grinder. Per 10 mg of liver, 100 µl of lysis buffer were used. Finally, the lysates were mixed with equal volumes of 10% SDS-containing sample buffer (final concentration, 5%) for gel electrophoresis.

Keratin-enriched fractions were prepared using the high salt extraction method described previously [1]. Briefly, pulverized liver tissue was homogenized with phosphate-buffered saline (PBS) containing 5 mM EDTA and 1% Triton-X 100 followed by centrifugation at 16,000 g. The supernatant, representing the cytosolic fraction, was mixed with SDS-containing sample buffer and later subjected to immunoblot analysis of soluble K8 levels. The pellet comprising cytoskeletal proteins was resuspended in High Salt buffer (10 mM Tris pH 7.5; 140 mM NaCl; 1.5 M KCl; 4 mM EDTA; 0.5% Triton X-100 and 0.1% SDS), incubated at 4°C for 30 minutes and repelleted. After washing the pellet with PBS containing 5 mM EDTA, the keratin-

enriched fraction was solubilized with 8 M urea and mixed with SDS-containing sample buffer.

Coomassie staining and immunoblotting. Protein lysates were separated by SDS-polyacrylamide gel electrophoresis (6−10%). Gels were either stained with Coomassie brilliant blue or proteins were transferred to nitrocellulose membranes. The membranes were blocked with 5% milk powder in PBS with 0.1% Tween-20 and subsequently incubated with primary antibodies followed by incubation with HRP-conjugated secondary antibodies. Detection was carried out using SuperSignal® West Pico Chemiluminescent Substrate (Thermo Scientific, Rockford, IL) and a Fusion FX chemiluminescence system (PEQLAB, Erlangen, Germany). Quantification of protein bands was performed using QuantiScan version 1.5 software (Biosoft, Cambridge, UK).

Expression and purification of recombinant proteins. C-terminally GST-tagged PRDs of murine epiplakin were bacterially expressed and affinity-purified as described before [2].

Blot overlay of liver keratins with plakin repeat domains. After gel electrophoresis, liver keratin extracts were immobilized to a nitrocellulose membrane which was subsequently cut between each protein lane. Each membrane stripe was separately incubated with PBS containing 0.1% Tween-20 and 5 µg/ml of individual recombinant GST-tagged epiplakin PRDs. Immunodetection of bound proteins was performed using a GST-antibody. Keratins were visualized using mouse monoclonal antibodies (mAbs) recognizing K8 and K18, respectively.

Keratin PRD-GST Pulldown*.* Primary hepatocytes were lysed with pre-chilled 2% Empigen BB (Calbiochem, San Diego, CA) in PBS, supplemented with 5 mM EDTA, 0.1 mM PMSF, Complete Mini protease inhibitor tablets (Roche) and Phosphatase Inhibitor Cocktails 2 and 3 (Sigma) for 1 hour at 4°C. The cell lysates were cleared by centrifugation at 16,000 g for 20 minutes, followed by incubation of the supernatants with recombinant GST-tagged epiplakin PRDs coupled to glutathione-Sepharose 4B beads (GE Healthcare, Chalfont St. Giles, UK) over night at 4°C. Beads were washed extensively with PBS and finally resuspended in 10% SDS-containing sample buffer for elution of the bound proteins which were subsequently analyzed via immunoblotting using antibodies to K8.

Antibodies*.* The following primary antibodies were used for immunoblotting (IB), immunofluorescence microscopy (IFM) and immunohistochemistry (IHC): mAbs to K8 (Ks8.7, Progen; IB), K18 (Ks18.04, Progen; IB), GST (clone GST-2, Sigma-Aldrich), K8 pS73 (LJ4) [3], desmoplakin (DP I/II 236.23.1, Progen, Heidelberg, Germany; IFM), occludin (OC-3F10, Life Technologies, Carlsbad, CA; IFM), and e-cadherin (36/E-Cadherin, BD Biosciences, San Jose, CA; IFM); rat mAbs to K8 (Troma I, Developmental Studies Hybridoma Bank, University of Iowa, Iowa City, IA [4]; IB, IFM, IHC), and K19 (Troma III, Developmental Studies Hybridoma Bank, University of Iowa [4]; IF); rabbit mAb to K19 (Epitomics, Inc., Burlingame, CA, IHC); affinity-purified rabbit antibodies to epiplakin (IB, IFM, IHC) [5], K18 pS33 (8250) [6], cleaved caspase-3 (Asp 175) (Cell Signaling; IFM), ubiquitin (Sigma; IFM), and Hsp70 (R&D Systems, Minneapolis, MN; IFM); guinea pig serum to p62 (GP62-C, Progen; IF). Secondary antibodies were donkey anti-rat rhodamine red (RRX)-conjugated IgGs (IFM), donkey anti-rabbit IgGs conjugated to Alexa Fluor 488 (A-488) (IFM), donkey anti-guinea pig RRX-conjugated IgGs (IFM), goat anti-mouse/rabbit/rat horseradish

peroxidase (HRP)-conjugated IgGs (IB) (all from Jackson Immunoresearch, West Grove, PA); rabbit anti-rat (IHC) and goat anti-rabbit biotin-conjugated IgGs (IHC) (both from Dako).

Isolation and cultivation of primary hepatocytes*.* Primary hepatocytes from wild-type (WT) and *Eppk1*$^{-/-}$ mice were isolated by a two-step collagenase perfusion and enriched by differential centrifugation using a Percoll gradient (Sigma, St Louis, MO). Hepatocytes were seeded on collagen-coated dishes.

Bile acid treatment of primary hepatocytes*.* 18 hours after seeding, primary hepatocytes isolated from WT and *Eppk1*$^{-/-}$ livers were incubated with serum-free medium containing different concentrations of either chenodeoxycholic acid (CDCA; 50, 100 and 200 µM; Sigma) or taurocholic acid sodium salt hydrate (TCA; 1,200 and 1,800 µM; Sigma). As CDCA had to be pre-diluted in ethanol, medium comprising this bile acid also contained up to a maximum of 0.2% ethanol. Thus, medium with the corresponding ethanol concentration was used as negative control ruling out cell death induced by ethanol exposure alone. 24 hours after the addition of bile acids or ethanol, hepatocyte viability was assessed using the Live/Dead cell double staining kit (Sigma-Aldrich). For each data set, more than 5,000 cells from 3 individual isolations were counted.

Okadaic acid treatment of primary hepatocytes*.* Primary hepatocytes isolated from WT and *Eppk1*$^{-/-}$ livers were cultivated in the presence or absence of the low molecular weight chaperone trimethylamine N-oxide (TMAO; Sigma; 100 µM) for 18 hours before being incubated with serum-free medium containing either okadaic acid (OA; Calbiochem; 30 nM) in combination with TMAO (100 µM) or OA alone. After 60

minutes of OA exposure, cells were either lysed with SDS sample buffer or fixed with methanol and stained with an antibody recognizing K8. Subsequently, analysis of the IF network was performed followed by quantification of cells comprising filamentous or granular keratins. In total, more than 2500 cells from 3 individual isolations were counted for each experimental group.

Overexpression of fluorescently tagged proteins in primary hepatocytes. Primary hepatocytes were seeded on collagen-coated dishes as a mixed culture of *Eppk1*$^{-/-}$ and WT cells, in the presence or absence of TMAO (100 µM) and instantly transfected with a plasmid coding for full-length human K8 C-terminally tagged with EYFP or the same vector coding for EYFP only, using the Nanofectin transfection kit from PAA (PAA, Pasching, Austria). After 24 hours, the cells were fixed with formaldehyde and immunofluorescently labeled for epiplakin to distinguish between WT and *Eppk1*$^{-/-}$ cells. EYFP fluorescence from the K8-EYFP fusion protein was used to assess the pattern of exogenous keratins and K8-EYFP- and EYFP-positive cells were counted. To correct for variations in transfection rate between WT and *Eppk1*$^{-/-}$ hepatocytes in individual isolations, the number of K8-EYFP-positive cells per mm^2 was divided by the number of EYFP-positive cells per mm^2 for each genotype and single experiment. In total, 6 individual hepatocyte isolations were performed. 318 WT and 140 *Eppk1*$^{-/-}$ cells transfected with K8-EYFP as well as 359 WT and 446 *Eppk1*$^{-/-}$ hepatocytes transfected with EYFP were counted. In the rescue experiments with TMAO between 123 and 300 cells from 3 individual isolations were counted for each experimental group.

To study the cellular localization of single plakin-repeat domains (PRDs) of epiplakin, WT hepatocytes were transfected with pEGFP-N2 vectors (Clontech Laboratories,

Inc., Mountain View, CA) containing individual cDNAs coding for PRDs 1–9 and 16 [2]. To analyze potential colocalization of PRDs with keratin filaments, cells were fixed with methanol 24 hours after transfection and stained with an antibody recognizing K8.

Supplementary Figures

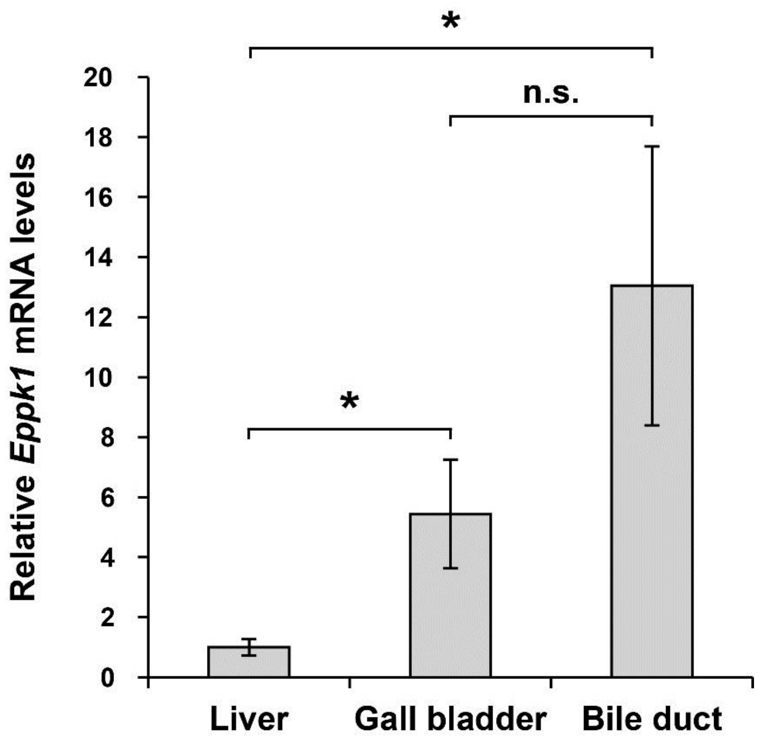

Fig. S1. Epiplakin transcript levels are higher in the biliary epithelium than in the liver. qRT-PCR analysis demonstrates significantly higher epiplakin mRNA levels in common bile duct and gall bladder compared to liver. Data are expressed as mean ± SEM; n = 6; *, $p < 0.05$; n.s., not significant.

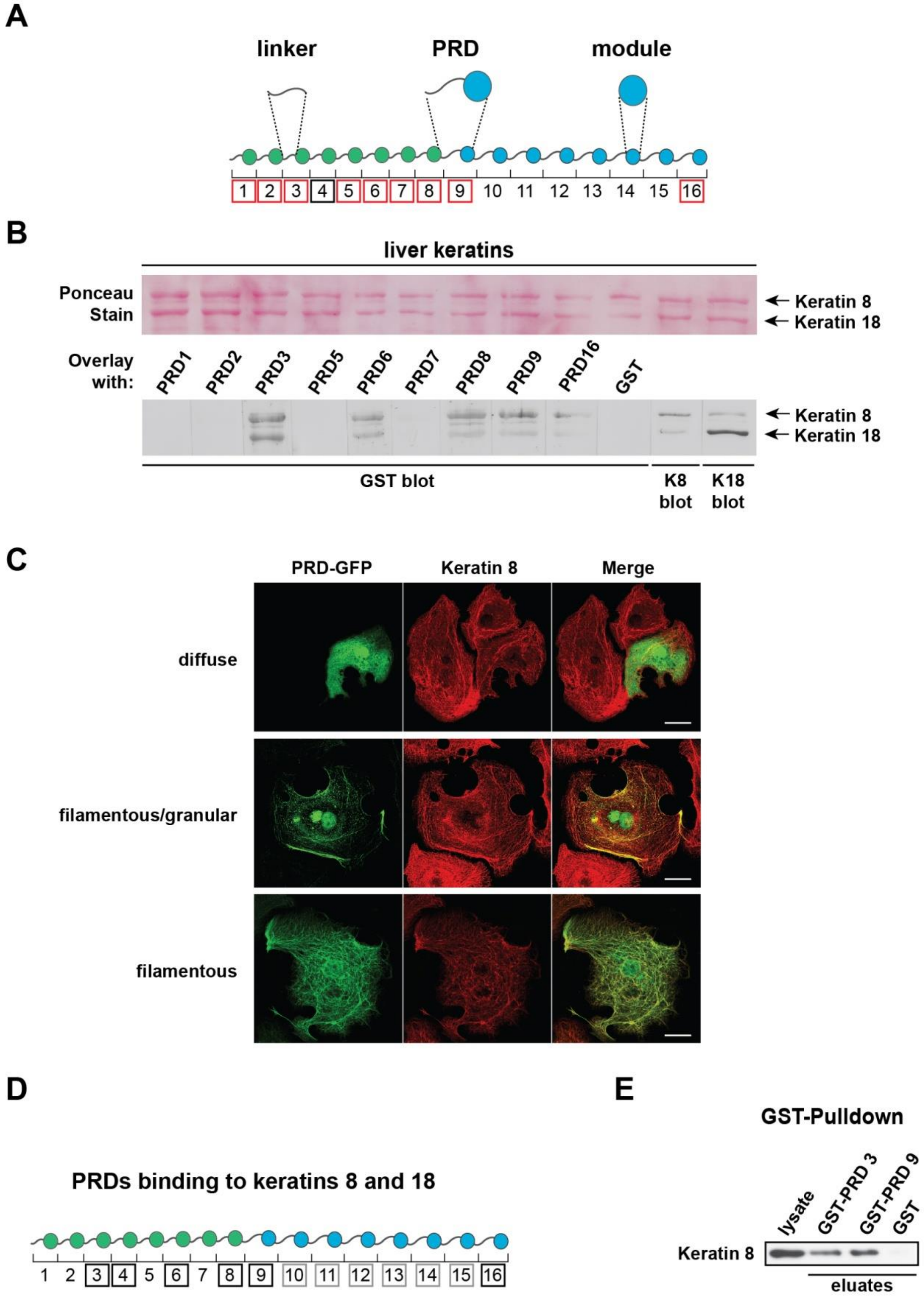

Fig. S2. Epiplakin interacts with hepatic keratins via multiple plakin repeat domains. (A) Domain structure of mouse epiplakin showing 16 plakin repeat

domains (PRDs), linkers (curved lines) and modules (circles). Modules that are virtually identical in amino acid sequence are shown in blue, those with lower homology in green. PRDs tested for keratin binding in (B) and (C) are marked by red frames. PRD4 (framed in black) was tested for keratin binding only by transfection studies (see Table S1). (B) Blot overlay of keratins isolated from mouse livers with 5 µg/ml of the PRD-GST fusion protein indicated. Overlay of K8 and K18 with GST was used as negative control. Ponceau Red stain demonstrates equal loading of hepatic keratins. Proteins bound to K8 and K18 were detected using a GST antibody whereas the position of keratins was visualized using antibodies recognizing K8 and K18 (arrows). (C) Primary hepatocytes were transfected with plasmids encoding EGFP fused to the different PRDs indicated in (A) followed by fixation 24 hours after transfection. Cells were subsequently stained with a K8 antibody and analyzed for their PRD distribution patterns and their potential colocalization with K8. Representative images of cells displaying diffuse, granular or filamentous PRD localization are shown. Scale bars, 20 µm. (D) Schematic drawing illustrating the findings obtained from experiments performed in (B) and (C). The domain structure of mouse epiplakin is depicted and PRDs shown to bind to K8 and K18 are marked by black frames. Given that PRD9 is representative for PRDs10–15 as they are identical in amino acid sequence, the keratin-binding properties of PRD9 were also ascribed to PRDs10–15 (marked by gray frames). (E) Co-sedimentation of K8 with two epiplakin PRD-GST fusion proteins. Proteins from a primary hepatocyte lysate and those that bound to epiplakin-GST-sepharose beads (eluates) were analyzed by immunoblotting using antibodies to K8; GST coupled to sepharose beads was used as negative control.

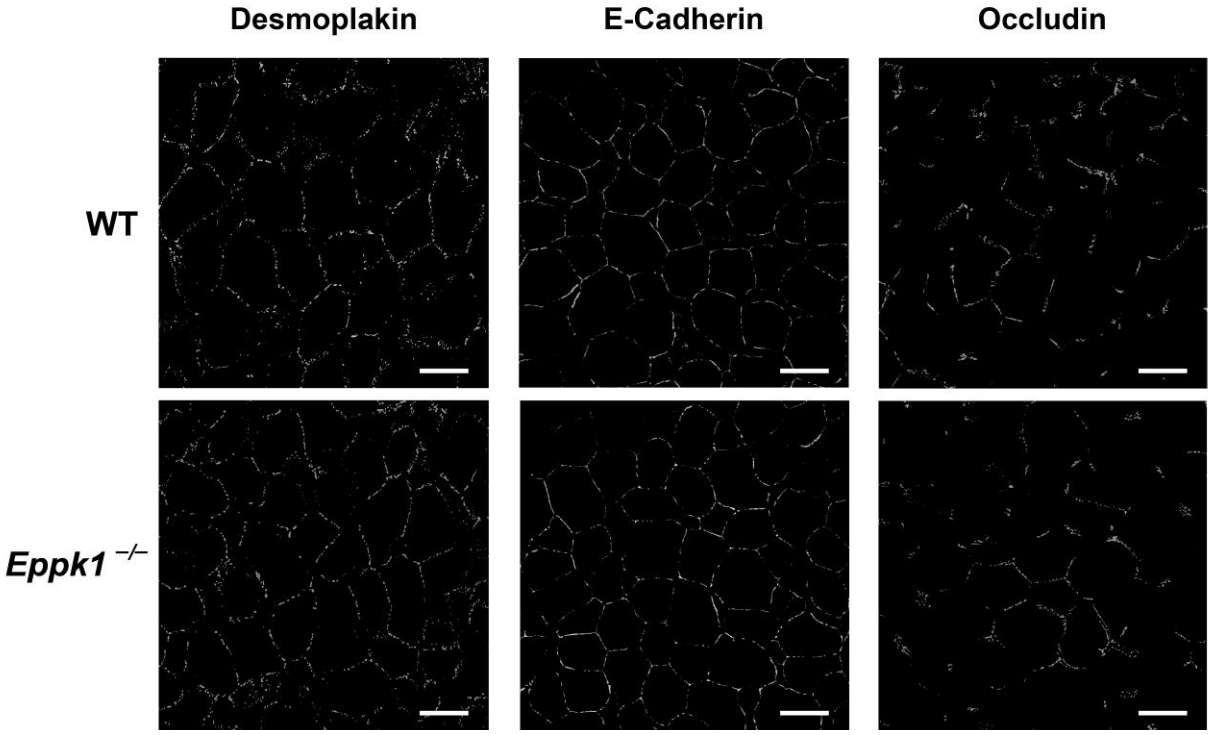

Fig. S3. Localization of the cell-cell junctional proteins desmoplakin, e-cadherin and occludin is unaltered in Eppk1$^{-/-}$ livers. Analysis of cell junctions in WT and *Eppk1*$^{-/-}$ livers using IFM. Antibodies recognizing desmoplakin, e-cadherin and occludin were used to analyze the morphology of desmosomes, adherens junctions and tight junctions, respectively. No irregularities in localization or staining intensity of these junctional proteins were detected in unstressed livers of *Eppk1*$^{-/-}$ mice. Scale bars, 20 µm.

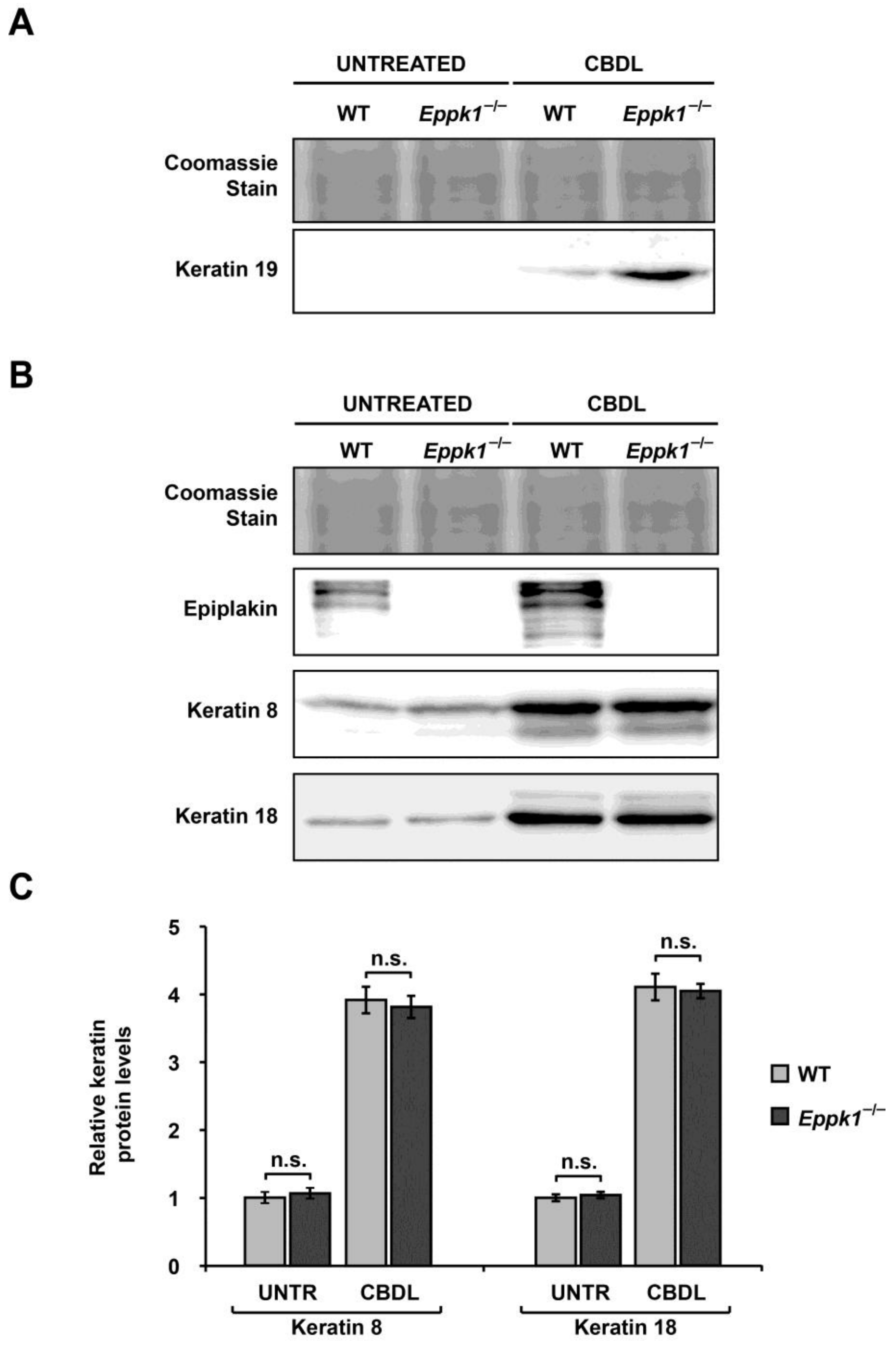

Fig. S4. Immunoblot analyses of CBDL-induced liver injury in WT and Eppk1$^{-/-}$ mice. (A) Representative immunoblot visualizing protein levels of K19 in livers of *Eppk1*$^{-/-}$ and WT mice before (untreated) and 5 days after CBDL. Robustly elevated K19 levels reflect enhanced ductular proliferation in *Eppk1*$^{-/-}$ livers. Coomassie staining demonstrates equal loading of protein lysates. (B) Representative immunoblots visualizing protein levels of epiplakin, K8 and K18 in livers of *Eppk1*$^{-/-}$

and WT mice before (untreated) and 5 days after CBDL. Coomassie staining demonstrates equal loading of protein lysates. Note that immunodetection of epiplakin results in several bands due to degradation of the protein during preparation of lysates. (C) Densitometric analysis of hepatic K8 and K18 levels of untreated and CBDL-treated mice. The untreated WT mean was arbitrarily set to 1. Note that keratin levels are similar in WT and *Eppk1*$^{-/-}$ samples before and after stress-induced upregulation of protein levels. Data are expressed as mean ± SEM; n = 3; n.s., not significant. UNTR, untreated.

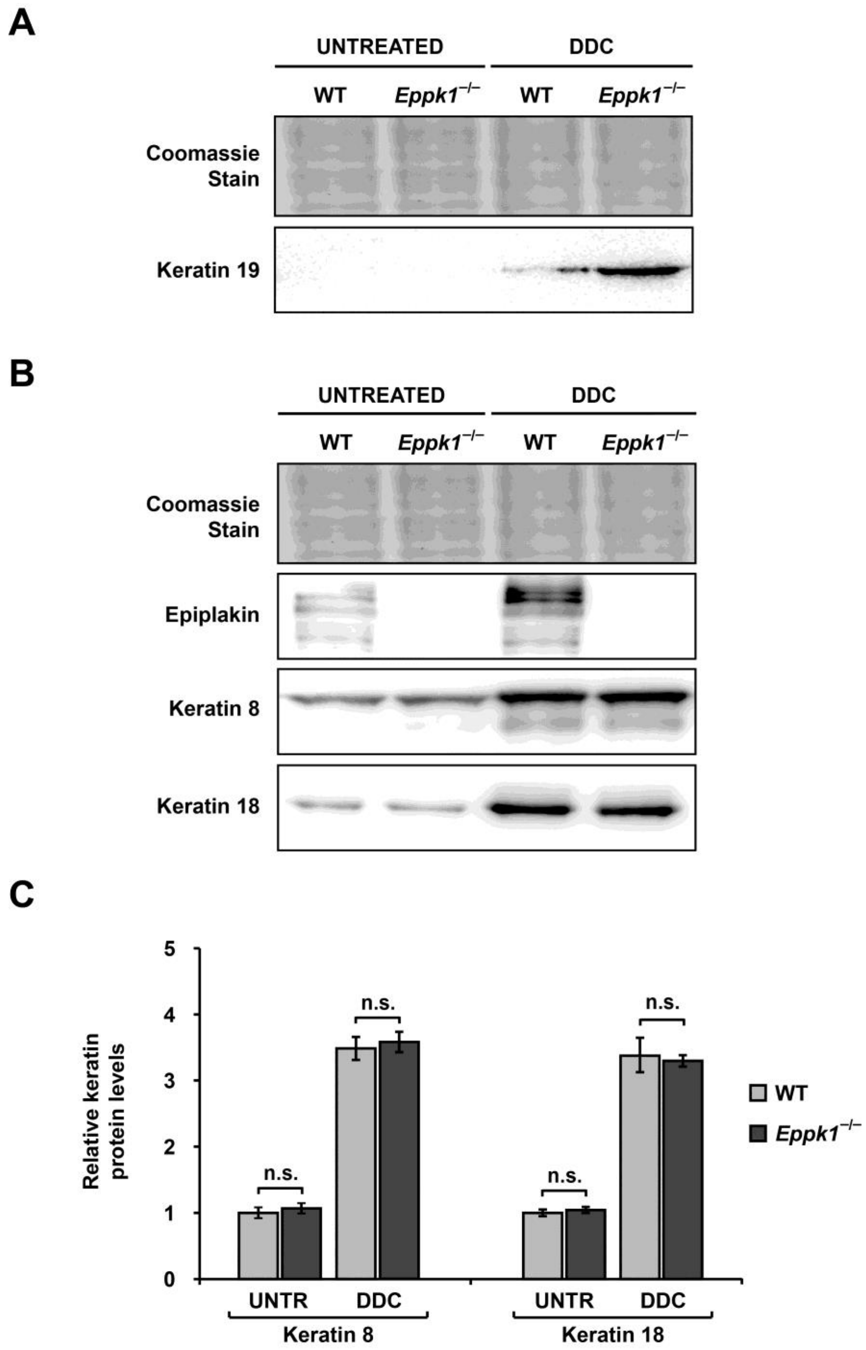

Fig. S5. Immunoblot analyses of DDC-induced liver injury in WT and Eppk1$^{-/-}$ mice. (A) Representative immunoblot visualizing protein levels of K19 in livers of *Eppk1*$^{-/-}$ and WT mice before (untreated) and after DDC treatment. Robustly elevated K19 levels reflect enhanced ductular proliferation in *Eppk1*$^{-/-}$ livers. Coomassie staining demonstrates equal loading of protein lysates. (B) Representative immunoblots visualizing protein levels of epiplakin, K8 and K18 in

livers of *Eppk1*$^{-/-}$ and WT mice before (untreated) and after DDC treatment. Coomassie staining demonstrates equal loading of protein lysates. Note that immunodetection of epiplakin results in several bands due to degradation of the protein during preparation of lysates. (C) Densitometric analysis of hepatic K8 and K18 levels of untreated and DDC-treated mice. The untreated WT mean was arbitrarily set to 1. Note that keratin levels are similar in WT and *Eppk1*$^{-/-}$ samples before and after stress-induced upregulation of protein levels. Data are expressed as mean ± SEM; n = 3; n.s., not significant. UNTR, untreated.

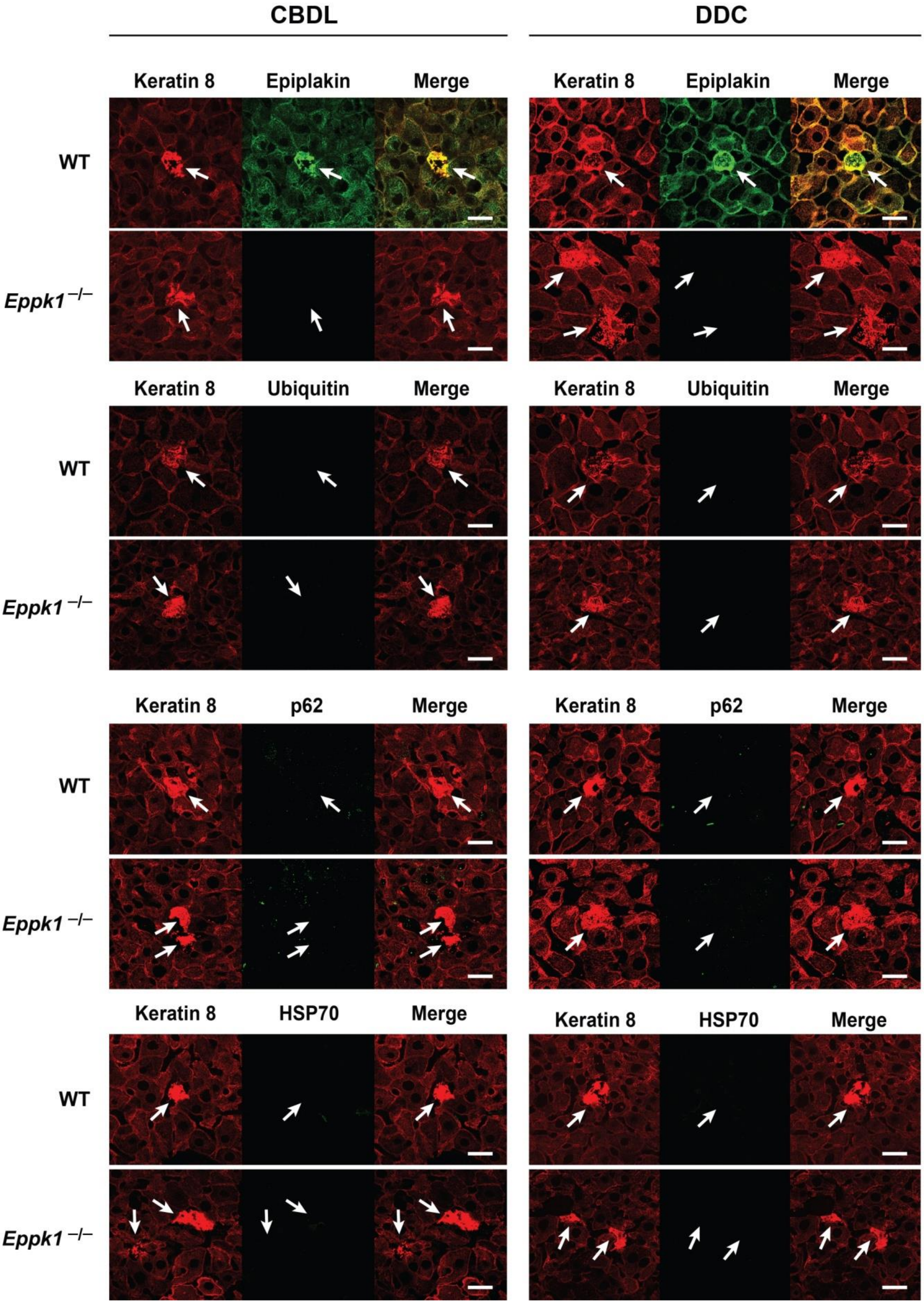

Fig. S6. Keratin aggregates formed after CBDL and DDC feeding do not colocalize with ubiquitin, p62 or Hsp70. IFM displaying representative hepatocytes

comprising keratin aggregates (arrows) as counted during statistical analysis in Figures 3D and 4C. Paraffin sections of CBDL- (left panel) and DDC-treated (right panel) livers isolated from WT and *Eppk1*$^{-/-}$ mice were co-stained with antibodies recognizing K8 as well as epiplakin, ubiquitin, p62 or Hsp70, respectively. Note that K8 aggregates detected in WT livers are epiplakin-positive, whereas all K8 granules were negative for ubiquitin, p62 or Hsp70, respectively. Scale bars, 20 µm.

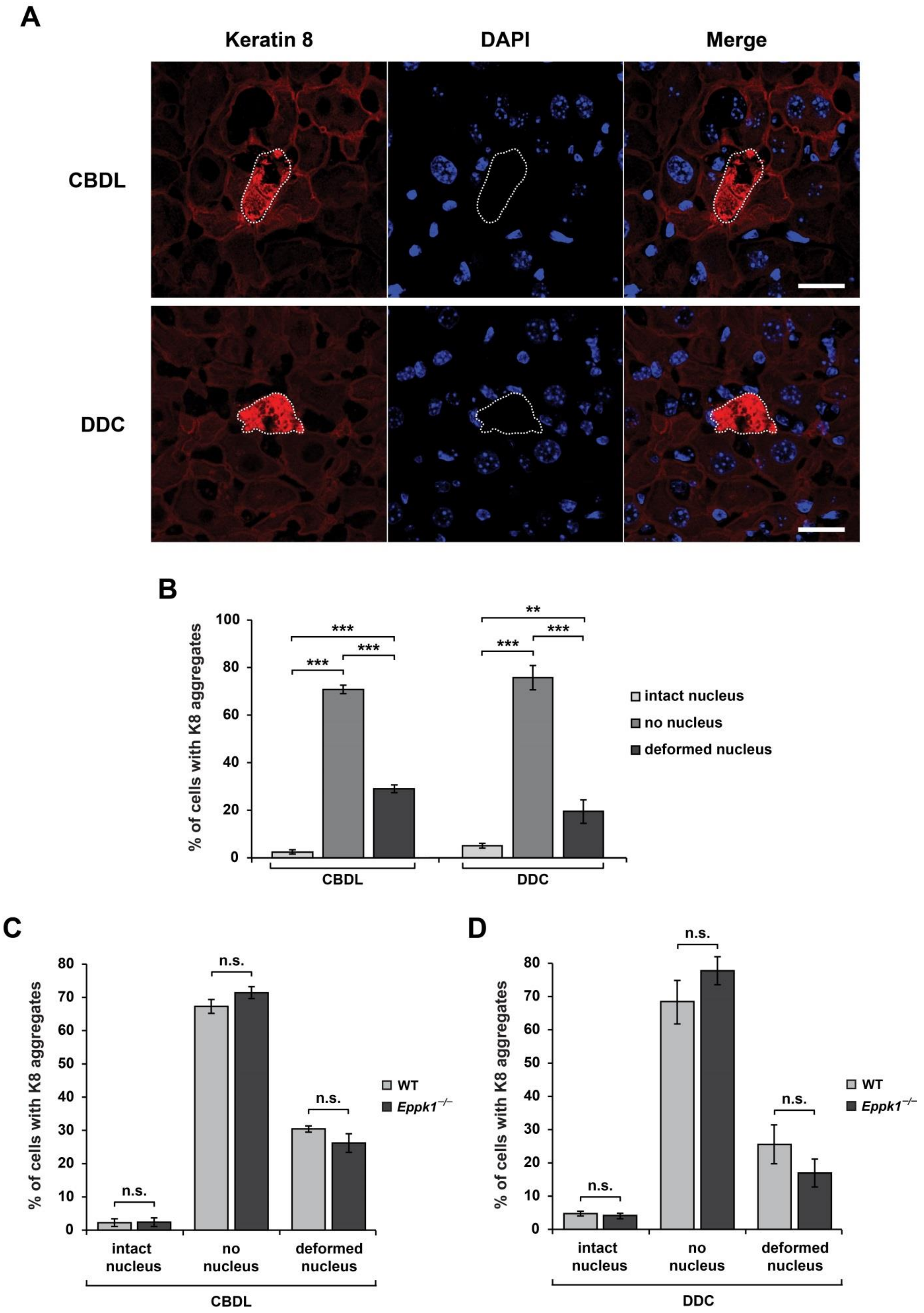

Fig. S7. Hepatocytes comprising keratin aggregates found in CBDL- and DDC-treated livers predominantly display loss of nuclei. (A) Liver paraffin sections

derived from WT and *Eppk1*$^{-/-}$ mice subjected to CBDL or DDC treatment were immunofluorescently labeled with antibodies recognizing K8. Using DAPI staining, hepatocytes comprising keratin aggregates found in CBDL and DDC livers of both genotypes (dashed lines) were examined with regard to their nuclear staining pattern. Representative hepatocytes displaying keratin granules and lacking nuclei are shown. Scale bars, 20 µm. (B) Statistical analysis of presence and shape of nuclei in hepatocytes comprising keratin aggregates found in CBDL and DDC livers of both genotypes demonstrates complete loss of nuclear structures in most evaluated cells. Data are expressed as mean ± SEM; $n = 6$; **, $p < 0.01$; ***, $p < 0.0005$. (C) Statistical analysis of presence and shape of nuclei in hepatocytes comprising keratin aggregates found in WT and *Eppk1*$^{-/-}$ CBDL livers showing no difference between both genotypes. Data are expressed as mean ± SEM; $n = 3$; n.s., not significant. (D) Statistical analysis of presence and shape of nuclei in hepatocytes comprising keratin aggregates found in WT and *Eppk1*$^{-/-}$ DDC livers showing no difference between both genotypes. Data are expressed as mean ± SEM; $n = 3$; n.s., not significant.

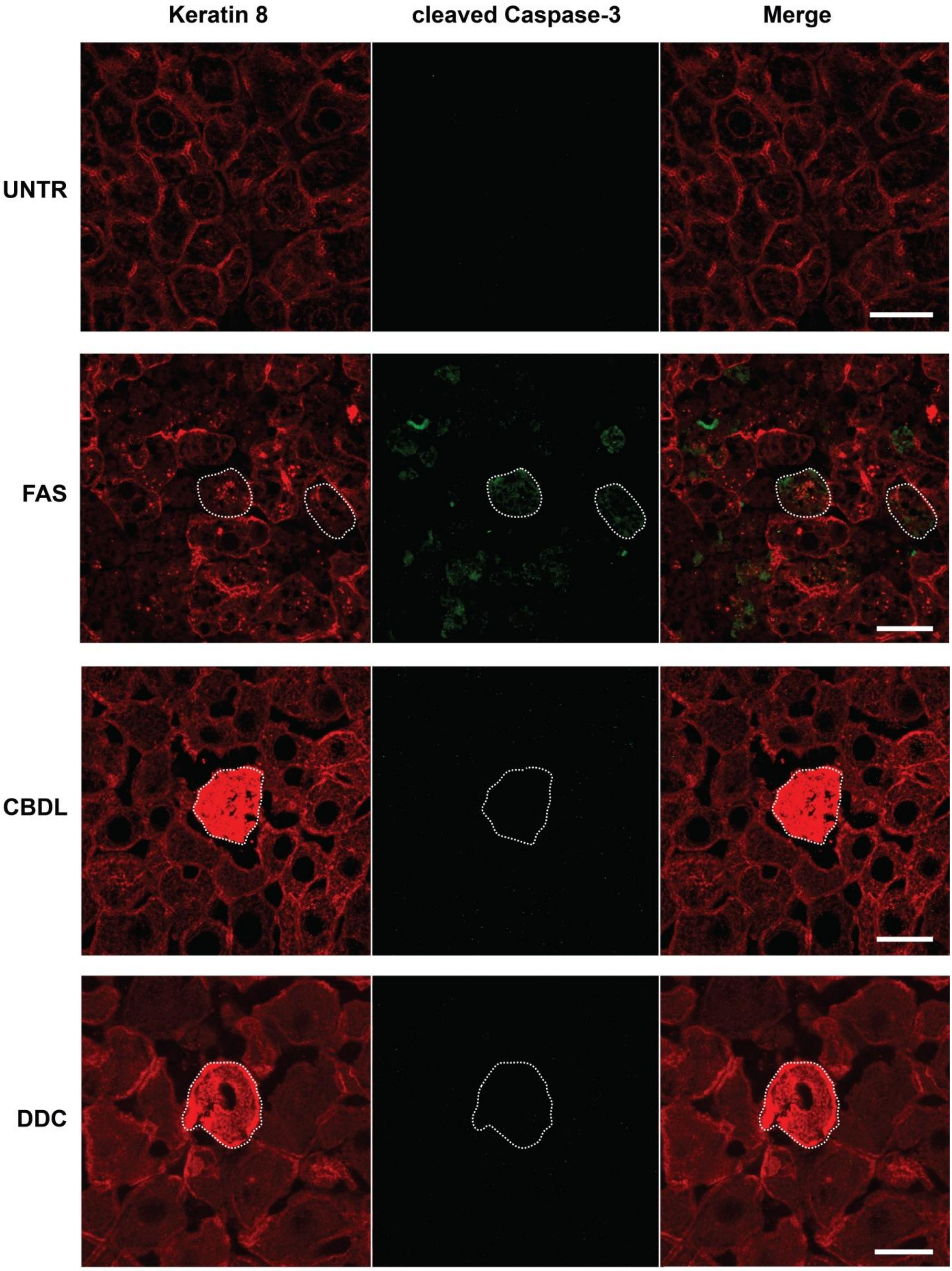

Fig. S8. Death of hepatocytes comprising keratin aggregates is not caused by activation of apoptotic pathways. Immunostainings of liver paraffin sections

derived from healthy mice (UNTR) and mice subjected to intraperitoneal injection of anti-Fas agonistic antibody Jo2 (0.35 µg/g; 6 h), CBDL or DDC treatment. Liver paraffin sections were immunofluorescently labeled with antibodies recognizing K8 and cleaved caspase-3. For CBDL and DDC livers, representative hepatocytes comprising keratin aggregates as counted during statistical analysis in Figures 3D and 4C, respectively, are shown (dashed lines). While these cells are negative for cleaved caspase-3, this apoptotic marker is clearly detectable in hepatocytes displaying Fas-induced keratin reorganization (dashed lines). Scale bars, 20 µm.

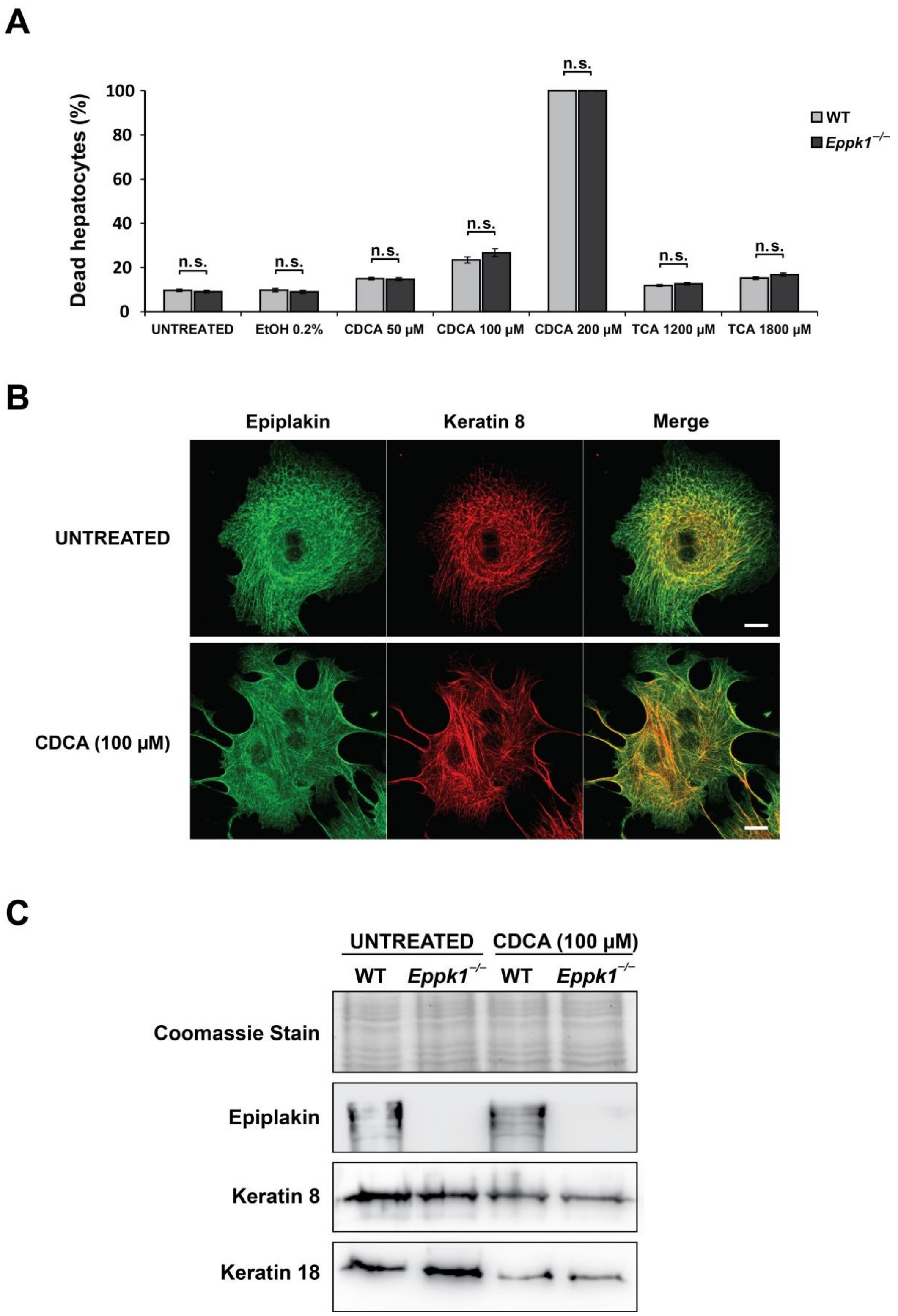

Fig. S9. Lack of epiplakin does not influence susceptibility of hepatocytes to bile acid exposure. (A) Measurement of hepatocyte viability after incubation with the bile acids chenodeoxycholic acid (CDCA) or taurocholic acid (TCA) used in the concentrations indicated. Note that medium containing 0.2% EtOH was used as

negative control for treatment with CDCA (solubilized in EtOH). 24 hours after bile acid addition, cell death was evaluated using simultaneous fluorescence staining of viable and dead hepatocytes labeled by an acetoxymethyl ester of calcein and propidium iodide solutions, respectively. Quantification of viable and dead cells revealed no differences in susceptibility of WT or *Eppk1*$^{-/-}$ hepatocytes to bile acid exposure. For each data set, more than 5,000 cells from 3 individual isolations were counted. Data are expressed as mean ± SEM; n.s., not significant. (B) Immunofluorescence microscopy depicting representative hepatocytes after 24 hours of incubation with serum-free medium (untreated) or serum-free medium containing 100 µM CDCA. Staining of hepatocytes with antibodies recognizing epiplakin and K8 demonstrates formation of thick keratin bundles but lack of keratin aggregates upon CDCA treatment. Scale bars, 10 µm. (C) Representative immunoblots visualizing protein levels of epiplakin, K8 and K18 in livers of *Eppk1*$^{-/-}$ and WT mice before (untreated) and 24 hours after CDCA treatment. The Coomassie stain demonstrates equal loading of protein lysates. Note that neither epiplakin nor keratin expression levels were upregulated upon bile acid exposure.

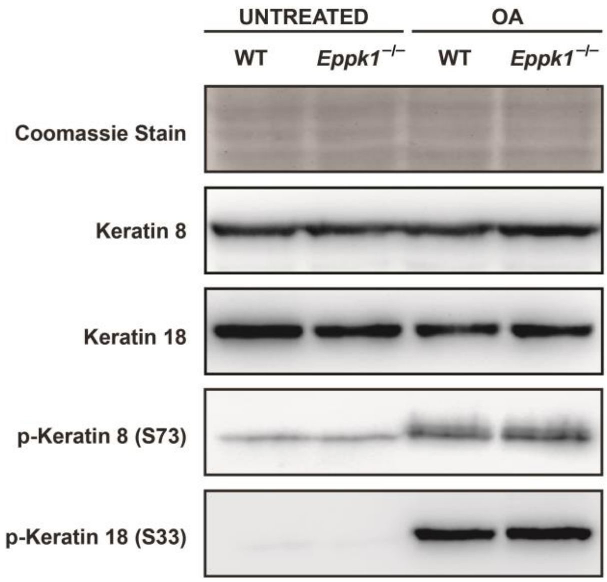

Fig. S10. Okadaic acid treatment results in comparable levels of phosphorylated keratins in WT and Eppk1$^{-/-}$ hepatocytes. Representative immunoblots visualizing protein levels of K8 and K18 as well as phospho-epitopes of K8 and K18 in *Eppk1*$^{-/-}$ and WT hepatocytes before (untreated) and after okadaic acid (OA) treatment. Coomassie staining demonstrates equal loading of protein lysates. Total K8 and K18 levels are comparable in WT and *Eppk1*$^{-/-}$ samples before and after OA treatment. Upon OA treatment, similar levels of K8 and K18 phosphorylated at S73 or S33, respectively, were detected in protein lysates of WT and *Eppk1*$^{-/-}$ hepatocytes.

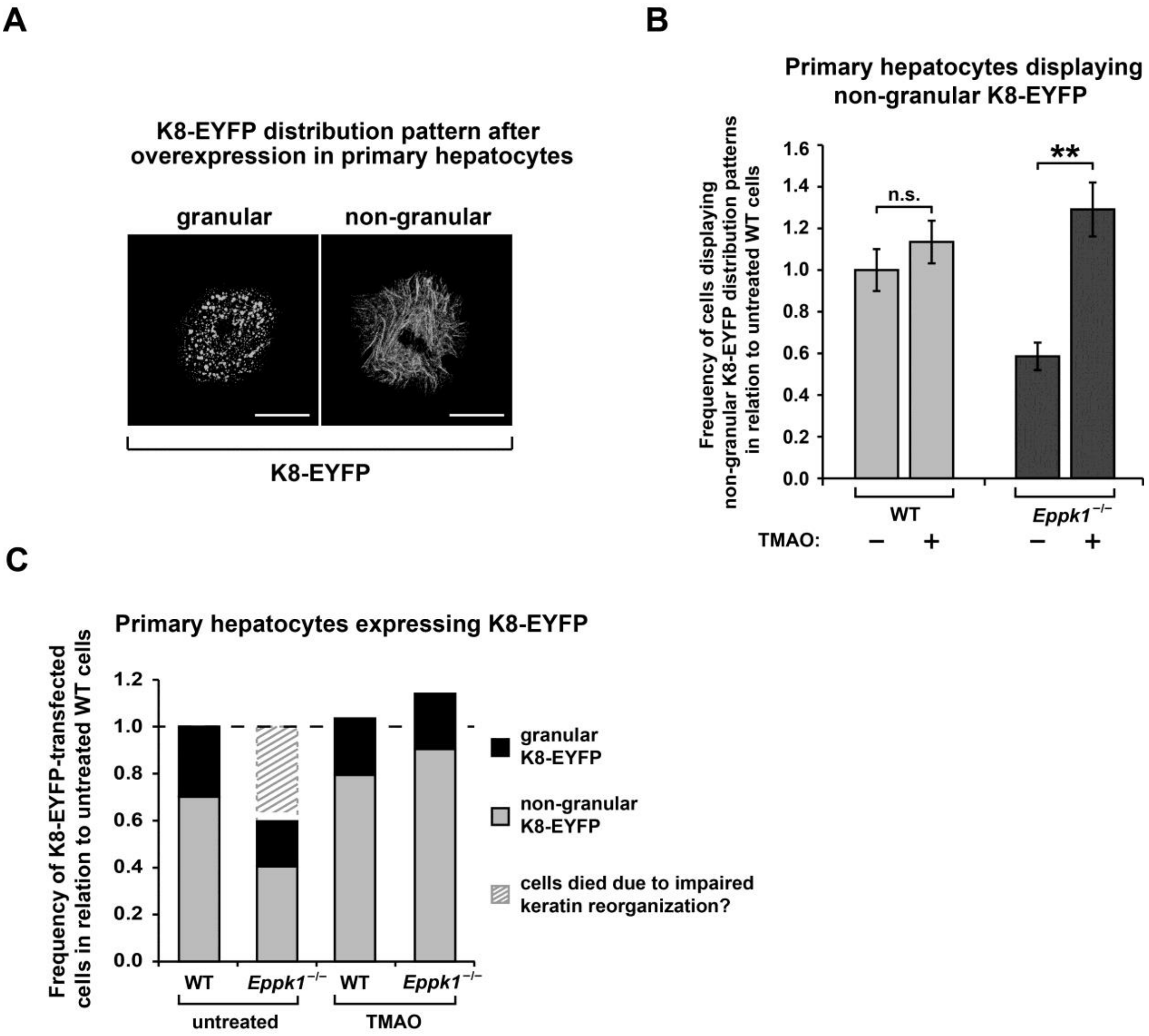

Fig. S11. TMAO treatment rescues the diminished number of Eppk1$^{–/–}$ hepatocytes displaying a non-granular K8-EYFP pattern. (A) Transfected primary hepatocytes displayed either granular or non-granular K8-EYFP-distribution patterns. Representative images of cells displaying a granular or a non-granular K8-EYFP network are shown. Scale bars, 30 µm. (B) Comparison of transfected WT and *Eppk1$^{–/–}$* cells comprising non-granular K8-EYFP distribution patterns. The untreated WT mean was arbitrarily set to 1. Incubation with TMAO significantly increased the number of *Eppk1$^{–/–}$* cells displaying a non-granular K8-EYFP distribution pattern. For each experimental group, between 123 and 300 cells from 3 individual isolations were counted. Data are expressed as mean ± SEM; **, $p < 0.005$; n.s., not significant. (C) Schematic overview summarizing the effects of TMAO on WT and

Eppk1$^{-/-}$ hepatocytes transfected with K8-EYFP. The WT mean was arbitrarily set to 1. The proportion of cells displaying granular K8-EYFP distribution is shown in black. In the presence of the chaperone TMAO, significantly higher numbers of K8-EYFP-transfected *Eppk1*$^{-/-}$ cells were detected than in its absence. However, the chaperone had no effect on the number of *Eppk1*$^{-/-}$ cells comprising granular K8-EYFP but clearly increased the number of *Eppk1*$^{-/-}$ cells displaying a non-granular K8-EYFP distribution pattern. This finding suggests that many *Eppk1*$^{-/-}$ cells overexpressing K8-EYFP died of impaired keratin reorganization, which is illustrated in the figure (dashed box).

Table S1. Summary of findings obtained from blot overlay and transfection experiments

	Transfection Studies<br>appearance in primary hepatocytes				Overlay Assays
	diffuse	granular	filamentous	colocalization with K8	binding to K8 and K18
**PRD1**	++	–	–	**–**	**–**
**PRD2**	++	++	–	**–**	**–**
**PRD3**	–	–	+++	**+++**	**+**
**PRD4**	–	++	++	**++**	n.d.
**PRD5**	+++	–	–	**–**	**–**
**PRD6**	–	+	++	**++**	**+**
**PRD7**	++	–	–	**–**	**–**
**PRD8**	–	+	++	**++**	**+**
**PRD9–15**	–	–	+++	**+++**	**+**
**PRD16**	–	+	+++	**+++**	**+**

Each PRD is characterized based on its appearance after transfection into hepatocytes (left columns) and its binding affinity to hepatic keratins in overlay assays (right column). Note that PRD9 is representative for PRDs10–15 as they are identical in amino acid sequence. n.d., not determined.

Supplementary References

[1] Ku NO, Toivola DM, Zhou Q, Tao GZ, Zhong B, Omary MB. Studying simple epithelial keratins in cells and tissues. Methods Cell Biol 2004;78:489-517.

[2] Spazierer D, Raberger J, Gross K, Fuchs P, Wiche G. Stress-induced recruitment of epiplakin to keratin networks increases their resistance to hyperphosphorylation-induced disruption. Journal of cell science 2008;121:825-833.

[3] Liao J, Ku NO, Omary MB. Stress, apoptosis, and mitosis induce phosphorylation of human keratin 8 at Ser-73 in tissues and cultured cells. The Journal of biological chemistry 1997;272:17565-17573.

[4] Boller K, Kemler R, Baribault H, Doetschman T. Differential distribution of cytokeratins after microinjection of anti-cytokeratin monoclonal antibodies. European journal of cell biology 1987;43:459-468.

[5] Spazierer D, Fuchs P, Pröll V, Janda L, Oehler S, Fischer I, et al. Epiplakin gene analysis in mouse reveals a single exon encoding a 725 kDa protein with expression restricted to epithelial tissues. The Journal of biological chemistry 2003;278:31657-31666.

[6] Ku NO, Liao J, Omary MB. Phosphorylation of human keratin 18 serine 33 regulates binding to 14-3-3 proteins. The EMBO journal 1998;17:1892-1906.
